# Supplementary material for: Sepsis recognition tools in acute ambulatory care: associations with process of care and clinical outcomes in a service evaluation of an Emergency Multidisciplinary Unit in Oxfordshire
Source: BMJ Open. 2018 Apr 9;8(4):e020497. doi: 10.1136/bmjopen-2017-020497 (PMC5892763; doi:10.1136/bmjopen-2017-020497)
Supplement: Supplementary data [file bmjopen-2017-020497supp001.pdf]

Supplementary Material

Supplementary Table 1:

Data Field	*Data type*
**Demographics**	
Study ID	Numerical
Age	Numerical
Sex	Categorical
Weight	Numerical
Height	Numerical
**Initial Attendance Information**	
Date of first attendance	Date
Attendance time	Time
Was the patient accompanied	Boolean
Most senior clinician reviewing patient on initial attendance	Categorical
**Patient Background**	
Comorbidities	Free Text
Known Dementia	Boolean
**Presenting complaints**	
Presenting Complaint	Free Text
Confusion	Boolean
Delirium	Boolean
SOB	Boolean
Confusion / altered behaviour	Boolean
Decreased mobility	Boolean
Increased care needs	Boolean
Falls	Boolean
Chest Pain, tachycardia, or palpitations	Boolean
Other respiratory (cough, wheeze, sore throat)	Boolean
Abdominal symptoms (pain, bloating, diarrhoea, nausea, vomiting, constipation	Boolean
Urinary or GU symptoms (dysuria, catheter problems, retention)	Boolean
Fatigue, weight loss, reduced oral intake	Boolean
Bilateral leg swelling	Boolean
Pain (back, hip/groin, shoulder, knee, wrist, toe, facial)	Boolean
Biochemical or haematological derangement (electrolytes, renal function, LFTs)	Boolean
Symptomatic anaemia	Boolean
Glycaemic derangement	Boolean
Other neurological (seizure, headache, tremor, visual symptoms, virtigo, tinnitus)	Boolean
PR bleed / malaena	Boolean
Leg weakness	Boolean
Fever	Boolean
Rash / erythema (incl face)	Boolean
Unilateral swelling	Boolean
Collapse	Boolean
Drowsiness	Boolean
Dizziness / unsteadiness	Boolean
Anxiety / low mood	Boolean
**Observations on Admission**	
Heart rate	Numerical
Systolic blood pressure	Numerical
Diastolic blood pressure	Numerical
Oxygen saturations	Numerical

Respiratory rate	Numerical
Temperature	Numerical
Postural drop in blood pressure	Boolean
AMTS	Ordinal
Glasgow coma score	Ordinal
**EMU Blood Results (categories)**	
Full blood count panel	Numerical
Urea and electrolytes panel	Numerical
C-reactive protein	Numerical
Liver function panel	Numerical
INR	Numerical
Lactate	Numerical
Troponin	Numerical
**Initial Diagnosis**	
Initial Diagnosis	Free text
Suspected infection	Boolean
Infection Source	Free text
**Treatments initiated**	
IV fluids	Boolean
Blood transfusion	Boolean
Nebulisers	Boolean
IV antibiotics	Boolean
Oral antibiotics	Boolean
IV iron	Boolean
IV furosemide	Boolean
Medications held	Boolean
Laxatives	Boolean
Analgesia	Boolean
Management	Free Text
**Outcomes**	
Initial plan (ambulation or admission)	Categorical
Escalation unit	Categorical
Documented reason for escalation	Free text
Date of death	Date
Discharge Date	Date
Number of visits to EMU	Numerical
Length of stay (if admitted)	Numerical
Date of EMU review	Date
Reason for EMU review	Free text
Unexpected re-admission within 30 days	Boolean

Legend:

Relevant variables collected as part of routine healthcare provision

Supplementary Table 2

Method	*Score components*		*Cut-off value*
	Available	*Unavailable*	
**NICE-HR** [1]			
	HR > 130 bpm	Mottled Skin	≥1
	RR > 25 min^{-1}	Cyanosis	
	New oxygen requirement	Non-blanching Rash	
	Systolic BP <90 mmHg	Urine Output	
	New Confusion	Systolic BP 40mmHG below normal	
**NICE-MR** [1]			
	HR 90 – 130 bpm	Steroid use / impaired immunity	≥1
	RR 21-24 min^{-1}	Trauma or Invasive Procedure	
	Systolic BP 90-100 mmHg	Redness/swelling/discharge at surgical site or breakdown of wound	
	Temperature <36°C		
	Reduced mobility or deterioration		
**qSOFA** [2]			
	GCS < 15	-	≥1
	RR > 21 min-1		
	Systolic BP ≤100 mmHg		
**SIRS** [3]			
	WCC <4.0 x10^3 or >12 x 10^3	-	≥2
	HR > 90 bpm		
	Temperature <36°C or >38°C		
	RR > 20 min^{-1}		
**NEWS** [4]			
	HR (multiple thresholds)	-	>4
	RR (multiple thresholds)		
	Oxygen Saturation (multiple thresholds)		
	Supplemental Oxygen (multiple thresholds)		
	Temperature (multiple thresholds)		
	Systolic BP (multiple thresholds)		
	Level of Consciousness (AVPU Scale)		

Legend

Components of diagnostic and severity assessment methods available and unavailable for use in this study. Cut-off values used as the threshold for predicting sepsis or poor patient outcome in this study. BPM – beats per minute, GCS – Glasgow Coma Scale, HR – Heart Rate, RR – Respiratory Rate, WCC – White Cell Count.

Supplementary Table 3:

Outcome Measure	*Test*	*PPV (%)*	*NPV (%)*	*Sensitivity (%)*	*Specificity (%)*
Escalated care					
	SIRS	55.6 (39.3 to 71.8)	80.8 (72.0 to 89.5)	57.1 (40.7 to 73.5)	79.7 (70.9 to 88.6)
	NICE High-Risk	40.4 (27.0 to 53.7)	77.4 (67.0 to 87.8)	60.0 (43.8 to 76.2)	60.8 (50.0 to 71.5)
	Nice Moderate- and High-Risk	31.0 (21.9 to 40.1)	71.4 (47.8 to 95.1)	88.6 (78.0 to 99.1)	12.7 (5.3 to 20.0)
	qSOFA	35.0 (24.5 to 45.5)	79.4 (65.8 to 93.0)	80.0 (66.7 to 93.3)	34.2 (23.7 to 44.6)
	NEWS >4	53.3 (35.5 to 71.2)	77.4 (68.4 to 86.3)	45.7 (29.2 to 62.2)	82.3 (73.9 to 90.7)
30-Day Mortality					
	SIRS	19.4 (6.5 to 32.4)	92.3 (86.4 to 98.2)	53.8 (26.7 to 80.9)	71.3 (62.5 to 80.1)
	NICE High-Risk	13.5 (4.2 to 22.7)	90.3 (83.0 to 97.7)	53.8 (26.7 to 80.9)	55.4 (45.8 to 65.1)
	Nice Moderate- and High-Risk	13.0 (6.4 to 19.6)	100.0 (100.0 to 100.0)	100.0 (100.0 to 100.0)	13.9 (7.1 to 20.6)
	qSOFA	13.8 (6.2 to 21.3)	94.1 (86.2 to 100.0)	84.6 (65.0 to 100.0)	31.7 (22.6 to 40.8)
	NEWS >4	20.0 (5.7 to 34.3)	91.7 (85.8 to 97.6)	46.2 (19.1 to 73.3)	76.2 (67.9 to 84.5)
30-Day Admission					
	SIRS	88.9 (78.6 to 99.2)	32.1 (21.7 to 42.4)	37.6 (27.3 to 47.9)	86.2 (73.7 to 98.8)
	NICE High-Risk	80.8 (70.1 to 91.5)	30.6 (19.2 to 42.1)	49.4 (38.8 to 60.0)	65.5 (48.2 to 82.8)
	Nice Moderate- and High-Risk	75.0 (66.5 to 83.5)	28.6 (4.9 to 52.2)	88.2 (81.4 to 95.1)	13.8 (1.2 to 26.3)
	qSOFA	77.5 (68.3 to 86.7)	32.4 (16.6 to 48.1)	72.9 (63.5 to 82.4)	37.9 (20.3 to 55.6)
	NEWS >4	93.3 (84.4 to 100.0)	32.1 (22.2 to 42.1)	32.9 (22.9 to 42.9)	93.1 (83.9 to 100.0)
30-Day Composite					
	SIRS	88.9 (78.6 to 99.2)	30.8 (20.5 to 41.0)	37.2 (27.0 to 47.4)	85.7 (72.8 to 98.7)
	NICE High-Risk	80.8 (70.1 to 91.5)	29.0 (17.7 to 40.3)	48.8 (38.3 to 59.4)	64.3 (46.5 to 82.0)
	Nice Moderate- and High-Risk	76.0 (67.6 to 84.4)	28.6 (4.9 to 52.2)	88.4 (81.6 to 95.1)	14.3 (1.3 to 27.2)
	qSOFA	77.5 (68.3 to 86.7)	29.4 (14.1 to 44.7)	72.1 (62.6 to 81.6)	35.7 (18.0 to 53.5)
	NEWS >4	93.3 (84.4 to 100.0)	31.0 (21.1 to 40.8)	32.6 (22.7 to 42.5)	92.9 (83.3 to 100.0)

Legend:

Analysis of Scoring Systems vs outcome measures in patients >85 years old with suspected infection (n=114). PPV = Positive predictive value, NPV = negative predictive value. PPV, NPV, Sensitivity, and Specificity show percentage and 95% confidence intervals.

Supplementary Figure 1. ROC Curves showing the discriminating ability of the different sepsis scoring systems for escalated care for infection

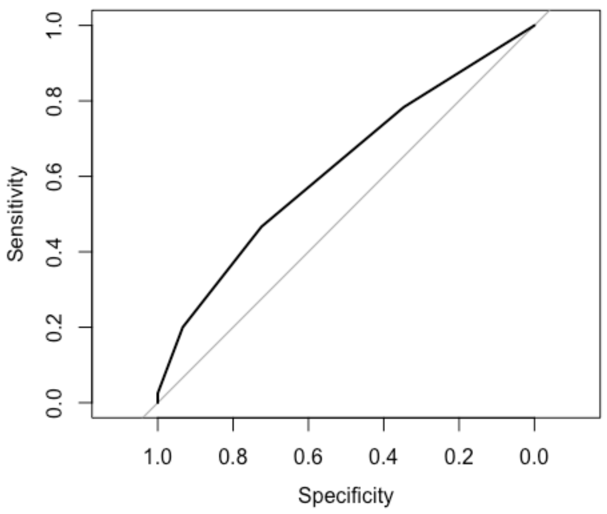

1a SIRS Area Under Curve (AUC) = 0.623 (95% CI: 0.5607-0.6843)

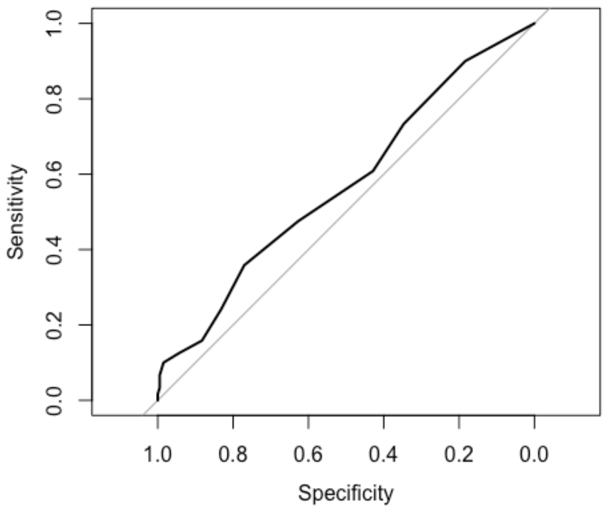

1b NEWS AUC = 0.573 (95% CI: 0.5079-0.6375)

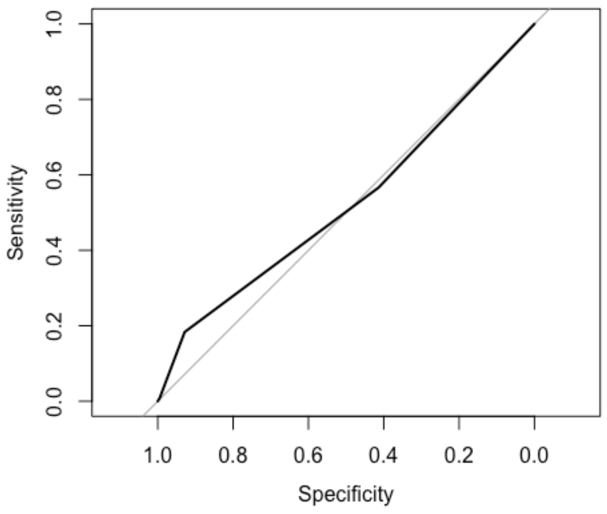

1c qSOFA AUC = 0.523 (95% CI: 0.4612-0.5855)

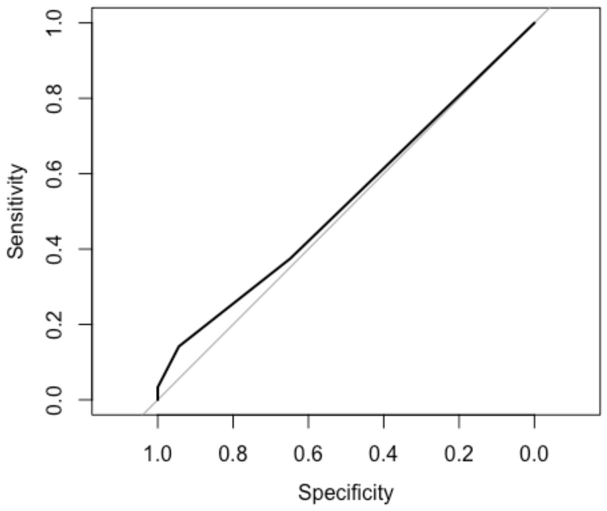

1d NICE High Risk AUC = 0.527 (95% CI: 0.4696-0.584)

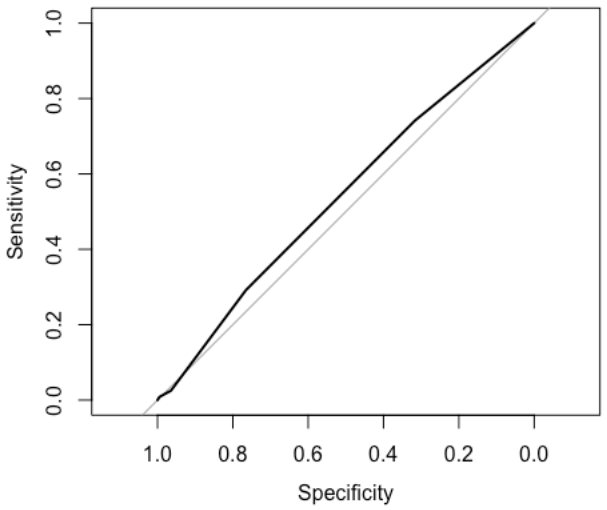

1e NICE Moderate and High Risk AUC = 0.540 (95% CI: 0.4784-0.6006)
