# Supplementary material for: Sepsis recognition tools in acute ambulatory care: associations with process of care and clinical outcomes in a service evaluation of an Emergency Multidisciplinary Unit in Oxfordshire
Source: BMJ Open. 2018 Apr 9;8(4):e020497. doi: 10.1136/bmjopen-2017-020497 (PMC5892763; doi:10.1136/bmjopen-2017-020497)
Supplement: Author's manuscript [file bmjopen-2017-020497.draft_revisions.pdf]

BMJ Open

BMJ Open is committed to open peer review. As part of this commitment we make the peer review history of every article we publish publicly available.

When an article is published we post the peer reviewers' comments and the authors' responses online. We also post the versions of the paper that were used during peer review. These are the versions that the peer review comments apply to.

The versions of the paper that follow are the versions that were submitted during the peer review process. They are not the versions of record or the final published versions. They should not be cited or distributed as the published version of this manuscript.

BMJ Open is an open access journal and the full, final, typeset and author-corrected version of record of the manuscript is available on our site with no access controls, subscription charges or pay-per-view fees (http://bmjopen.bmj.com).

If you have any questions on BMJ Open's open peer review process please email info.bmjopen@bmj.com

Sepsis recognition tools in acute ambulatory care: associations with process of care and clinical outcomes in a service evaluation

Journal:	*BMJ Open*
Manuscript ID	bmjopen-2017-020497
Article Type:	Research
Date Submitted by the Author:	07-Nov-2017
Complete List of Authors:	Camm, Christian; University of Oxford, Radcliffe Department of Medicine<br>Hayward, Gail; University of Oxford, Nuffield Department of Primary Care Health Sciences<br>Elias, Tania; Oxford University Hospitals NHS Foundation Trust, Geratology<br>Bowen, Jordan; Oxford University Hospitals NHS Foundation Trust, Geratology<br>Hassanzadeh, Roya; Imperial College London, Public Health and Primary Care<br>Fanshawe, Thomas; University of Oxford, Nuffield Department of Primary Care Health Sciences<br>Pendlebury, Sarah; Stroke Prevention Reseacrh Unit, Nuffield Department of Clinical Neurosciences<br>Lasserson, Daniel; University of Birmingham, Institute of Applied Health Research; University of Oxford, Nuffield Department of Medicine
**Primary Subject Heading**:	Health services research
Secondary Subject Heading:	Diagnostics, General practice / Family practice, Infectious diseases
Keywords:	EPIDEMIOLOGY, GENERAL MEDICINE (see Internal Medicine), GERIATRIC MEDICINE, Quality in health care < HEALTH SERVICES ADMINISTRATION & MANAGEMENT

Sepsis recognition tools in acute ambulatory care: associations with process of care and clinical outcomes in a service evaluation

C Fielder Camm[1], Gail Hayward[2], Tania C N Elias[3,4], Jordan S T Bowen[3,4], Roya Hassanzadeh[3,5], Thomas Fanshawe[2], Sarah Pendlebury,[4,6] Daniel Lasserson[7,8]

[1]Radcliffe Department of Medicine, University of Oxford, Oxford, UK

[2]Nuffield Department of Primary Care Health Sciences, University of Oxford, Oxford, UK

[3]Emergency Multidisciplinary Unit, Abingdon Hospital, Oxford Health NHS Foundation Trust, Oxford, UK

[4]Department of Geratology, Oxford University Hospitals NHS Foundation Trust, Oxford, UK

[5]Department of Public Health and Primary Care, Imperial College London, London, UK

[6]Nuffield Department of Clinical Neuroscience, University of Oxford, Oxford UK.

[7]Nuffield Department of Medicine, University of Oxford, Oxford, UK

[8]Institute of Applied Health Research, College of Medical and Dental Sciences, University of Birmingham, Birmingham, UK

Correspondence to:

Prof Daniel Lasserson, Professor of Ambulatory Care, Murray Learning Centre, Institute of Applied Health Research, College of Medical and Dental Sciences, University of Birmingham, Edgbaston, Birmingham, B15 2 TT. D.S.Lasserson@bham.ac.uk

Manuscript Word Count: 2,779 Words

For peer review only

Abstract

Objective

To assess the performance of currently available sepsis recognition tools in patients referred to a community based acute ambulatory care unit.

Design

Service evaluation of consecutive patients over a four month period.

Setting

Community based acute ambulatory care unit

Data collection and outcome measures:

Observations, blood results, and outcome data were analysed from patients with a suspected infection. Clinical features at first assessment were used to populate sepsis recognition tools including: systemic inflammatory responses syndrome (SIRS) criteria, national early warning score (NEWS), quick sequential organ failure assessment (qSOFA), and National Institute for Health and Care Excellence (NICE) criteria. Scores were assessed against the clinical need for escalated care (use of IV antibiotics, fluids, ongoing ambulatory care or hospital treatment) and poor clinical outcome (all-cause mortality and readmission at 30 days after index assessment).

Results

Of 533 patients (median age 81 years), 316 had suspected infection with 120 patients requiring care escalated beyond simple community care. SIRS had the highest positive predictive value (52.3%, 95% confidence interval (CI) [41.8% to 62.9%]) and negative predictive value (70.3%, 95% CI [63.1% to 77.5%]) for the need for escalated care. Both NEWS and SIRS were better at predicting the need for escalated care than qSOFA and NICE criteria in patients with suspected infection (all $p<0.001$). While new onset confusion predicted the need for escalated care for infection in patients ≥85 years old (n= 114), 23.7% of patients ≥85 years had new onset confusion without evidence for infection.

Conclusions

Acute ambulatory care clinicians should use caution in applying the new NICE endorsed criteria for determining need for intravenous therapy and hospital based location of care. NICE criteria have poorer performance when compared against NEWS and SIRS and new onset confusion was prevalent in patients aged ≥ 85 years without infection.

For peer review only

Strengths and limitations of this study

- Acute ambulatory care is applicable to an increasingly prevalent healthcare delivery model
- Consecutive patient evaluation to minimise selection bias
- Not all elements of the sepsis recognition models were available for scoring

Key Words

General Medicine, Geriatric Medicine, Quality in Healthcare, Epidemiology

For peer review only

Introduction

Sepsis, defined as 'life-threatening organ dysfunction caused by dysregulated host response to infection',[1] has a high mortality[2] but early recognition allows for prompt and effective treatment.[3] Therefore, tools for accurate recognition are needed at the earliest possible point after patients attend an initial healthcare assessment setting.

The Third International Consensus (SEPSIS-3) advocates the Sequential Organ Failure Assessment (SOFA)[1] score to evaluate sepsis. However, SOFA is not practical to use in the community and the Quick Sequential Organ Failure Assessment (qSOFA) score has been developed for use in rapid triage and in settings without access to blood tests.[4] The National Institute of Health and Care Excellence (NICE) has recently introduced new guidance regarding sepsis, including a tool for the diagnosis and triage of patients with suspected sepsis in community settings.[2] The use of the qSOFA, and the NICE high-risk and moderate risk criteria (NICE-HR and NICE-MR respectively) are a significant departure from the older systemic inflammatory response syndrome (SIRS) criteria.[5] In addition to sepsis recognition, the National Early Warning Score (NEWS) has been advocated by the Royal College of Physicians to track the clinical condition of patients presenting acutely for hospital and community assessment.[6]

New tools for sepsis recognition have not been evaluated in all healthcare environments that provide initial clinical assessments. Lower sensitivity or specificity of either qSOFA or NICE criteria compared with existing tools for detecting sepsis at initial assessment, particularly in the community, could result in poorer care overall. Lower sensitivity results in missed cases of sepsis compared with current recognition tools and lower specificity results in the unnecessary escalation of care including rapid transfer from community settings to emergency departments, unnecessary use of broad spectrum antibiotics and rapid fluid administration.

The performance of newer recognition scores, such as qSOFA and the NICE criteria, may vary with clinical setting. Low specificity of NICE criteria has been suggested by the large number of acute medical unit admissions it detects as needing rapid assessment within one hour, in comparison with qSOFA and SIRS.[7 8] Furthermore, among emergency department patients with low qSOFA scores, a significant proportion require critical care interventions which would not have been anticipated using qSOFA[9] and the now abandoned SIRS criteria have shown more accurate prediction of death than qSOFA in some emergency department cohorts.[10] Given that in intensive care unit patients, SOFA has a better performance than SIRS,[11 12] it is likely that the clinical setting and nature of the patient cohorts in which these tools are applied have a strong influence on their accuracy to detect sepsis and stratify patients based on risk of poor outcomes.

The acute ambulatory care model, which is recommended as a strategy in acute medicine to meet increasing demands for care within constrained resources,[13] represents a particular challenge for the recognition of sepsis. Whilst ambulatory care is becoming increasingly prevalent,[14] there are very few tools designed to support clinical decision making in the ambulatory care environment. Patients who are deemed suitable for ambulatory care do not have very disordered

For peer review only - http://bmjopen.bmj.com/site/about/guidelines.xhtml

physiology[15], even though there may be an emerging underlying significant disease process. Furthermore, identification of sepsis in older patients with frailty is complex as acute functional decline has many other causes,[16] yet mortality and morbidity related to sepsis is higher in the older population.[17] One of the critical decisions in ambulatory care management of suspected infection is selecting which patients can be safely discharged with oral therapy and follow up from their general practitioner and which patients are at higher risk of clinical deterioration and require care that is enhanced, either through an ambulatory platform with daily parenteral antibiotics with senior clinical review, or medical admission.

The ambulatory care clinician, faced with the diagnostic and management challenge of early sepsis recognition and the need to safely manage patients on an ambulatory pathway, can choose from four different sepsis recognition tools (SIRS, NEWS, NICE-HR/MR, and qSOFA). This study aims to evaluate the performance of these tools by examining their association with processes of care and clinical outcomes in patients with suspected infection in an acute ambulatory care setting. Given the uncertainty about application to older people in whom there is a wide differential diagnosis for acute frailty syndromes such as confusion, we undertook a subgroup analysis in patients 85 years or older.

For peer review only

Methods

Using a structured clerking proforma including a short cognitive test (abbreviated mental test score, AMTS)) as described previously,[13] the direct care team collected healthcare data as part of routinely provided healthcare from consecutive patients presenting to Abingdon Hospital's Emergency Multidisciplinary Unit (EMU) run by Oxford Health NHS Foundation Trust between 12/08/2015 and 08/12/2015. Patients were included if they were resident within Oxfordshire, allowing for appropriate follow-up details to be obtained, and had a clinical phenotype suggesting possible infection, in keeping with the recommended application of sepsis screening tools.[5] The use of routine healthcare data collected by the direct care team for the purposes of determining adherence to national guidance and application of clinical prediction tools to inform future service development was prospectively approved as a service evaluation by Oxford University Hospitals NHS Foundation Trust (providers of the medical care on EMU), with audit datix reference number 3812, and permission to publish was granted by Oxford Health NHS Foundation Trust R&D Department.

Study setting:

The EMU model of care provides assessment and treatment for adults with acute illness where an ambulatory treatment path is considered likely by the initial contact healthcare provider. Facilities include point of care blood testing (electrolytes, renal function, blood gases, lactate, C-reactive protein, troponin, INR), plain X Ray, and intravenous delivery of medications and fluids. Referrals are primarily from general practitioners and paramedic teams in the community.

Patients whose care needs cannot be met with an ambulatory pathway are admitted to either a community hospital or an associated acute hospital.

Process of care and clinical outcomes:

Details regarding the patients' presentation, investigations, treatment, and outcome were prospectively documented at the time of assessment on the EMU. Patients were considered to have a possible infection if a clinical suspicion of infection was recorded by the treating clinician or if the presenting complaint suggested an infective cause for their symptoms. Processes of care included use of intravenous (IV) antibiotics, IV fluids and pathway of care (ambulatory vs hospital) and re-admission within 30 days. Clinical outcome was 30-day all-cause mortality.

We tested the ability of sepsis scoring systems to select patients who require continued monitoring and treatment from the medical team, either via ambulatory or bed based pathways. Patients were considered to have required escalated care for an infection if *all* the following conditions were met: (1) the patient had a possible infection, (2) the patient received IV antibiotics and IV fluids, (3) the patient was either admitted to an inpatient facility or subsequently brought back to the EMU for ≥1 daily reviews, or enrolled in the 'hospital @ home' programme to receive ongoing IV antibiotics and daily review after initial EMU assessment. Furthermore, patients with suspected infection who did not meet conditions (2) and (3) were considered to have needed escalated care if they died within 7-days of review or required re-admission for infection within 30 days. This strategy was used as it collectively identifies patients for whom initial discharge from ambulatory care with no or oral antibiotics may have been inappropriate.

Sepsis Scoring Systems:

We evaluated the accuracy of SIRS,[18] qSOFA,[6] NEWS,[2] NICE-HR and NICE-MR from NICE Guideline NG51[7] for detecting the need for escalated care to treat suspected bacterial infection. Each method was undertaken in accordance with guidance provided by issuing bodies. Features associated with these tools available in our data-set and cut-off values for prediction of sepsis or poor outcome are detailed in Supplementary Table 1. For scoring systems in which some items were not collected in this dataset (NICE-MR), the score was calculated without use of these items. For cases in which individual items required for a score were missing, the score for that individual was calculated without that item, in line with clinical practice. During the analysis NICE-MR and HR were combined in some analyses to replicate the use of the moderate criteria in clinical practice.

Statistical Analysis:

Continuous variables are expressed as either mean (standard deviation) or median (interquartile range [IQR]) and compared across suspected infection/no suspected infection and escalated care/no escalated care groups using the independent student's T-test or Mann-Whitney U-test respectively. Categorical and ordinal variables are reported as frequency (percentage) and compared between groups using the χ^2 or Fisher's exact test. The diagnostic accuracy of each

For peer review only - http://bmjopen.bmj.com/site/about/guidelines.xhtml

predictive tool was assessed using positive predictive value (PPV), negative predictive value (NPV), sensitivity and specificity, with 95% confidence intervals. Predictive tools were compared with one another using McNemar's test, and we consider p-values of less than 0.005 to be statistically significant in these comparisons (based on a conservative Bonferroni correction for ten comparisons). The performance of sepsis tools was assessed in the whole dataset and in a subgroup of the oldest patients (≥85 years) in predicting the need for escalated care, mortality, and readmission within 30 days of index assessment. Analysis was performed using Version 9 of the SAS system for Windows. Copyright © 2002-2010 by SAS Institute Inc., Cary, NC, USA.

Results

A total of 533 patients were assessed during the observation time period. Ten patients were not local residents and did not have follow-up data and were excluded from the analysis (Figure 1). Demographic data of the study population are provided in Table 1. 316 patients were considered to have a potential infection with 120 patients requiring escalated treatment for infection. Features most significantly associated with need for escalated care were increased heart rate (89.6 (18.9) vs. 83.8 (17.8), p=0.006), increased temperature (37.0 (0.9) vs 36.6 (0.6), p<0.001), and increased white cell count (14.5 (17.4) vs. 9.3 (3.7), p=0.003). In the whole cohort, there was no association between new onset confusion and need for escalated care (22/120 (20.0%) vs. 27/196 (14.0%), p=0.172). A full list of features associated with escalated care is displayed in Table 1.

Of 186 patients ≥85 years old (45% of the cohort), 114 patients had a suspected infection as the cause for their presentation. Among patients ≥85 years with suspected infection, those who met criteria for escalated care (n=35, 30.7%) also demonstrated the positive findings for the whole cohort in terms of difference in physiological markers, but new confusion was significantly associated with escalated care in this subgroup (Table 1).

The PPV, NPV, sensitivity and specificity of each assessment tool against the need for escalated care, 30-day mortality, 30-day admission, and a composite marker of 30-day mortality or admission for the cohort with suspected infection are shown in Table 2. SIRS had the highest PPV (50.9%, 95% CI [41.6% to 60.3%]) and NPV (68.9%, 95% CI [62.6% to 75.3%]). Using McNemar's test, the combined high- and moderate-risk NICE criteria were shown to be significantly worse at predicting escalated care compared with SIRS (p<0.001), NEWS (p<0.001), and NICE High-risk criteria alone (p=0.004) (Table 3), a result driven by very low specificity of the combined high- and moderate-risk NICE criteria (21.4%, 95% CI [15.7 to 27.2]). Furthermore, both SIRS and NEWS were shown to have better performance against qSOFA (both p<0.001). NEWS and SIRS had the highest specificity for detecting 30-day mortality (67.0 95% CI [61.6 to 72.4] and 73.5 95% CI [68.5 to 78.6] respectively).

Among patients ≥85 years old, the PPV and NPV of both SIRS and NEWS were higher than NICE or qSOFA scores for escalated care and for the combined 30 day clinical outcome (Supplementary Table 2). Whilst NICE criteria had the highest

sensitivity at detecting poor clinical outcomes (mortality or admission within 30 days) (88.4%), this was at the cost of very low specificity (14.3%).

Discussion

Of the tools assessed, SIRS and NEWS were shown to have the highest PPV and NPV for escalated care in the dataset overall and in the subgroup of patients ≥ 85 years. Whilst NICE criteria and qSOFA had better performance in detecting requirement for escalated care in patients ≥85 years, their poorer specificity contributed to a lower PPV. The presence of new confusion was associated with escalated care among patients with suspected infection in the subgroup of patients aged ≥ 85 years.

Although SIRS was shown to be the most predictive of escalated care in patients with suspected infection in this study, the combined high and moderate risk NICE criteria would capture the most patients requiring escalated care but at the cost of very low specificity i.e. a high false positive rate. These results contrast with the previously suggested limitations in predictive ability for poor outcomes when compared against other potential sepsis recognition and stratification tools which have led to a move away from using SIRS.[5] This may reflect applicability in different patient cohorts as Kaukonen *et al.* demonstrated that 12.1% of intensive care unit patients with severe sepsis did not meet ≥2 SIRS criteria.[2 19 20] However, they also showed that those with SIRS positive sepsis had significantly increased mortality compared with those not meeting these criteria. Churpek *et al.* demonstrated that 47% of an acute patient cohort met ≥2 SIRS criteria during admission and concluded that screening patients for sepsis based on these criteria would be unwise.[19] However, this group did not select patients in whom infection was suspected and instead applied it to all admissions, irrespective of presenting complaint.

The importance of identifying sepsis in the initial stages of clinical contact to allow for prompt and effective treatment has been well documented.[20] However, our results have shown that those systems endorsed by both NICE and Sepsis-3 have limitations related to high false-positive rates as compared with both SIRS and NEWS, which could lead to substantial over-treatment.

The introduction of new-onset confusion as a predictor of sepsis in NICE guidance risks increasing the use of inappropriate antibiotics in the older patient with frailty. Although our results show a significant association between the development of new confusion and the need for escalated care among patients ≥ 85 years with infection, there was a high prevalence of new onset confusion in this age group without any evidence of underlying infection. While infection is a principle cause of delirium,[3] there is limited high-quality evidence that altered mental state in older adults is predictive of sepsis itself [21] and delirium has a wide range of potential causes in older people.[5]

For peer review only

Our data show that the new combined NICE high-risk and moderate-risk criteria are likely to significantly increase the number of patients that are determined as potentially having sepsis at initial assessment in ambulatory care. As such, this is likely to increase the number of patients receiving intravenous antibiotics which may conflict with the national and global priorities of antibiotic stewardship.[21] Furthermore, in the ambulatory care setting their introduction may increase conversion to formal hospital admission which our data suggest will not be justified based on the clinical outcomes we recorded.

Limitations

Several limitations of this work are acknowledged. Data collection for this work was undertaken at a time when SIRS was the recommended assessment tool for potential sepsis. As such, incorporation bias may explain some degree of the correlation between this method of assessment and the diagnosis and treatment of sepsis. However, SIRS was not calculated as part of the standard assessment proforma in this cohort and white cell count was not available at initial review as it is not part of the point of care testing portfolio. This limitation would not explain the correlation between SIRS and NEWS with mortality at 30-days.

Not all elements of the new NICE criteria for high- and moderate-risk sepsis were available for this analysis. However, the limited specificity of both these methods in determining sepsis would only be worsened by the addition of further criteria to this analysis. Furthermore, we argue that several of those unavailable elements (e.g. Mottled Skin, Cyanosis, and Urine Output) represent those that would either not be available in the acute ambulatory setting or are based on subjective assessment and therefore more likely to vary between clinicians.

Conclusions:

This study demonstrates that new NICE recommended sepsis tools do not outperform the existing tools in an ambulatory care setting in predicting the need for escalated care for infection and clinical outcomes at 30 days. Although NICE tools had higher sensitivity, this was at the expense of very low specificity. SIRS had the highest predictive values but the requirement of a white cell count implies that it can only be used widely if this is tested at the point of care. Further research should be undertaken to determine applicability of NICE sepsis guidance in different healthcare settings where acutely unwell patients are assessed before widespread adoption in the NHS.

For peer review only

Funding Statement

CFC is funded by an NIHR Academic Clinical Fellowship. The NIHR Oxford Biomedical Research Centre supported DSL and SP with grant number AC14/035 supporting JSTB and TCNE. JSTB is also supported by the NIHR Oxford Collaboration for Leadership in Applied Health Research and Care (CLAHRC) This article presents independent research funded by the National Institute for Health Research (NIHR) Diagnostic Evidence Co-operative Oxford. The opinions expressed in this study are those of the authors and not necessarily those of the NIHR, the Department of Health or the NHS.

Contribution Statement

CFC undertook analyses and drafted the initial manuscript. GH and TF advised on analytical methodology, interpreted data and critically revised the manuscript. JSTB, JCNE and RH designed data collection methods, collected data, clinically interpreted analyses and critically revised the manuscript. SP designed the service evaluation, data collection methods and critically revised the manuscript. DSL designed the service evaluation, advised on analytical methods, interpreted data and critically revised the manuscript.

Data sharing statement

No additional data are available

Conflict of Interest

None declared.

For peer review only

References

1. Fleischmann C, Scherag A, Adhikari NK, et al. Assessment of Global Incidence and Mortality of Hospital-treated Sepsis. Current Estimates and Limitations. *Am J Respir Crit Care Med* 2016;193(3):259-72. doi: 10.1164/rccm.201504-0781OC

2. Singer M, Deutschman CS, Seymour CW, et al. The Third International Consensus Definitions for Sepsis and Septic Shock (Sepsis-3). *JAMA* 2016;315(8):801-10. doi: 10.1001/jama.2016.0287

3. Ferrer R, Martin-Loeches I, Phillips G, et al. Empiric antibiotic treatment reduces mortality in severe sepsis and septic shock from the first hour: results from a guideline-based performance improvement program. *Crit Care Med* 2014;42(8):1749-55. doi: 10.1097/CCM.0000000000000330

4. Vincent JL, Moreno R, Takala J, et al. The SOFA (Sepsis-related Organ Failure Assessment) score to describe organ dysfunction/failure. On behalf of the Working Group on Sepsis-Related Problems of the European Society of Intensive Care Medicine. *Intensive Care Med* 1996;22(7):707-10.

5. National Institute of Health and Care Excellence. NICE Guideline 51 - Sepsis: recognition, assessment and early management: National Institute of Health and Care Excellence, 2016.

6. Bone RC, Balk RA, Cerra FB, et al. Definitions for Sepsis and Organ Failure and Guidelines for the Use of Innovative Therapies in Sepsis. *Chest* 1992;101(6):1644-55. doi: 10.1378/chest.101.6.1644

7. National Early Warning Score (NEWS). Standardising the assessment of acute-illness severity in the NHS: Royal College of Physicians (London), 2012.

8. Smith GB, Prytherch DR, Schmidt PE, et al. Review and performance evaluation of aggregate weighted 'track and trigger' systems. *Resuscitation* 2008;77(2):170-9. doi: 10.1016/j.resuscitation.2007.12.004

9. Thompson A, Stevens M, Collin I, et al. Evolving sepsis definitions and their impact on Acute Medical Units. *Acute medicine* 2017;16(1):25-29. [published Online First: 2017/04/21]

10. Moskowitz A, Patel PV, Grossestreuer AV, et al. Quick Sequential Organ Failure Assessment and Systemic Inflammatory Response Syndrome Criteria as Predictors of Critical Care Intervention Among Patients With Suspected Infection. *Crit Care Med* 2017 doi: 10.1097/ccm.0000000000002622 [published Online First: 2017/08/02]

11. Askim A, Moser F, Gustad LT, et al. Poor performance of quick-SOFA (qSOFA) score in predicting severe sepsis and mortality - a prospective study of patients admitted with infection to the emergency department. *Scandinavian journal of trauma, resuscitation and emergency medicine* 2017;25(1):56. doi: 10.1186/s13049-017-0399-4 [published Online First: 2017/06/11]

12. Williams JM, Greenslade JH, McKenzie JV, et al. Systemic Inflammatory Response Syndrome, Quick Sequential Organ Function Assessment, and Organ Dysfunction: Insights From a Prospective Database of ED Patients With Infection. *Chest* 2017;151(3):586-96. doi: 10.1016/j.chest.2016.10.057 [published Online First: 2016/11/24]

13. Commission FH. Future hospital: caring for medical patients. A report from the Future Hospital Commission to the Royal College of Physicians. London, 2013.

14. Nasa P, Juneja D, Singh O, et al. Severe sepsis and its impact on outcome in elderly and very elderly patients admitted in intensive care unit. *J Intensive Care Med* 2012;27(3):179-83. doi: 10.1177/0885066610397116

15. Raith EP, Udy AA, Bailey M, et al. Prognostic Accuracy of the SOFA Score, SIRS Criteria, and qSOFA Score for In-Hospital Mortality Among Adults With Suspected Infection Admitted to the Intensive Care Unit. *Jama* 2017;317(3):290-300. doi: 10.1001/jama.2016.20328 [published Online First: 2017/01/24]

16. Ala L, Mack J, Shaw R, et al. Selecting ambulatory emergency care (AEC) patients from the medical emergency in-take: the derivation and validation of the Amb score. *Clinical medicine (London, England)* 2012;12(5):420-6. [published Online First: 2012/10/30]

17. Girard TD, Opal SM, Ely EW. Insights into severe sepsis in older patients: from epidemiology to evidence-based management. *Clin Infect Dis* 2005;40(5):719-27. doi: 10.1086/427876

For peer review only

18. Pendlebury ST, Lovett NG, Smith SC, et al. Observational, longitudinal study of delirium in consecutive unselected acute medical admissions: age-specific rates and associated factors, mortality and re-admission. *BMJ Open* 2015;5(11):e007808. doi: 10.1136/bmjopen-2015-007808

19. Kaukonen KM, Bailey M, Pilcher D, et al. Systemic inflammatory response syndrome criteria in defining severe sepsis. *N Engl J Med* 2015;372(17):1629-38. doi: 10.1056/NEJMoa1415236

20. Churpek MM, Zadravecz FJ, Winslow C, et al. Incidence and Prognostic Value of the Systemic Inflammatory Response Syndrome and Organ Dysfunctions in Ward Patients. *Am J Respir Crit Care Med* 2015;192(8):958-64. doi: 10.1164/rccm.201502-0275OC

21. Fong TG, Tulebaev SR, Inouye SK. Delirium in elderly adults: diagnosis, prevention and treatment. *Nat Rev Neurol* 2009;5(4):210-20. doi: 10.1038/nrneurol.2009.24

For peer review only

Tables

Table 1:

Item	All patients with suspected infection				≥85 years old				<85 years old			
		Need for escalated care				Need for escalated care				Need for escalated care		
	N	Yes	No	p-value	N	Yes	No	p-value	N	Yes	No	p-value
Subjects	316	120 (38.9%)	196 (62.0%)	.	114	35 (30.7%)	79 (69.3%)	.	202	85 (42.1%)	117 (57.9%)	.
Proportion Male	316	57 (47.5%)	79 (40.3%)	0.210	114	10 (28.6%)	33 (41.8%)	0.180	202	47 (55.3%)	46 (39.3%)	0.024
Age (years)	316	74.5 (16.6)	75.2 (18.3)	0.708	114	90.2 (3.3)	89.7 (3.9)	0.442	202	68.0 (15.5)	65.4 (17.7)	0.283
Heart Rate (beats min^{-1})	315	89.6 (18.9)	83.8 (17.8)	0.006	114	89.5 (19.2)	79.29 (15.5)	0.003	201	89.6 (18.9)	86.9 (18.6)	0.306
Respiratory Rate	314	21.6 (6.4)	21.3 (5.3)	0.643	113	24.4 (8.2)	20.7 (5.2)	0.016	201	20.5 (5.1)	21.7 (5.4)	0.094
Systolic Blood Pressure	314	132.2 (20.1)	135.9 (26.7)	0.172	113	129.1 (21.4)	132.0 (26.1)	0.534	201	133.5 (19.5)	138.5 (26.9)	0.133
Oxygen Saturations (%)	313	96 (94 - 98)	97 (95 - 98)	0.007	113	96 (93 - 97)	97 (95 - 98)	0.009	200	96 (94 - 98)	97 (94 - 98)	0.132
Temperature (^{o}C)	314	37.0 (0.9)	36.6 (0.6)	<0.001	113	36.9 (0.9)	36.5 (0.6)	0.044	201	37.1 (0.9)	36.7 (0.6)	0.001
AMTS	303	10 (8 - 10)	10 (9 - 10)	0.155	107	7 (5 - 9)	9 (7 - 10)	0.008	196	10 (9 - 10)	10 (9 - 10)	0.532
Known Dementia Diagnosis	316	21 (17.5%)	23 (11.7%)	0.151	114	9 (25.7%)	14 (17.7%)	0.327	202	12 (14.1%)	9 (7.7%)	0.140
New Confusion	316	22 (20.0%)	27 (14.0%)	0.172	114	15 (48.4%)	18 (23.7%)	0.012	202	7 (8.9%)	9 (7.7%)	0.769
White Cell Count(x 10^9/L)	274	14.5 (17.4)	9.3 (3.7)	0.003	99	13.9 (6.4)	9.3 (4.1)	0.001	175	14.7 (20.4)	9.3 (3.3)	0.028
Mortality (30-Day)	316	14 (11.7%)	11 (5.6%)	0.053	114	8 (22.9%)	5 (6.3%)	0.010	202	6 (7.1%)	6 (5.1%)	0.567
Barthel Score ≥15	316	80 (66.7%)	150 (76.5%)	0.056	114	17 (48.6%)	53 (67.1%)	0.061	202	63 (74.1%)	97 (82.9%)	0.129

Legend:

Demographic characteristics of patients assessed on the EMU during this analysis with suspected infection (n=316). AMTS – Abbreviated Mental Test Score. Results displayed as N(%), mean (standard deviation), or median(inter-quartile range) unless otherwise indicated. For the scoring systems evaluated, all data points evaluated were available in the following numbers: SIRS (n=272), NICE High-Risk (n=314), NICE Moderate- and High-Risk (n=314), qSOFA (n=314), NEWS (n=313).

Table 2

Outcome Measure	*Test*	*PPV (%)*	*NPV (%)*	*Sensitivity (%)*	*Specificity (%)*
**Escalated care**					
	SIRS	50.9 (41.6 to 60.3)	68.9 (62.6 to 75.3)	46.7 (37.7 to 55.6)	72.4 (66.2 to 78.7)
	NICE High-Risk	39.5 (30.5 to 48.4)	62.9 (56.2 to 69.5)	37.5 (28.8 to 46.2)	64.8 (58.1 to 71.5)
	Nice Moderate- and High-Risk	38.4 (32.4 to 44.4)	63.6 (52.0 to 75.2)	80.0 (72.8 to 87.2)	21.4 (15.7 to 27.2)
	qSOFA	37.2 (30.2 to 44.2)	60.9 (52.6 to 69.2)	56.7 (47.8 to 65.5)	41.3 (34.4 to 48.2)
	NEWS >4	48.9 (38.4 to 59.3)	66.2 (60.1 to 72.4)	35.8 (27.3 to 44.4)	77.0 (71.2 to 82.9)
**30-Day Mortality**					
	SIRS	12.7 (6.5 to 19.0)	94.7 (91.6 to 97.7)	56.0 (36.5 to 75.5)	67.0 (61.6 to 72.4)
	NICE High-Risk	12.3 (6.3 to 18.3)	94.6 (91.4 to 97.7)	56.0 (36.5 to 75.5)	65.6 (60.2 to 71.1)
	Nice Moderate- and High-Risk	9.6 (5.9 to 13.3)	98.5 (95.5 to 100.0)	96.0 (88.3 to 100.0)	22.3 (17.6 to 27.1)
	qSOFA	11.5 (6.9 to 16.1)	97.0 (94.1 to 99.9)	84.0 (69.6 to 98.4)	44.3 (38.6 to 50.0)
	NEWS >4	12.5 (5.6 to 19.4)	93.9 (90.7 to 97.0)	44.0 (24.5 to 63.5)	73.5 (68.5 to 78.6)
**30-Day readmission**					
	SIRS	82.7 (75.7 to 89.8)	34.0 (27.5 to 40.4)	40.1 (33.7 to 46.5)	78.7 (70.1 to 87.2)
	NICE High-Risk	74.6 (66.6 to 82.6)	29.7 (23.4 to 36.0)	37.4 (31.1 to 43.7)	67.4 (57.7 to 77.2)
	Nice Moderate- and High-Risk	74.8 (69.4 to 80.2)	39.4 (27.6 to 51.2)	82.4 (77.4 to 87.3)	29.2 (19.8 to 38.7)
	qSOFA	74.9 (68.6 to 81.1)	32.3 (24.4 to 40.3)	60.4 (54.0 to 66.7)	48.3 (37.9 to 58.7)
	NEWS >4	78.4 (69.8 to 87.0)	30.7 (24.7 to 36.7)	30.4 (24.4 to 36.4)	78.7 (70.1 to 87.2)
**30-Day Composite**					
	SIRS	83.6 (76.7 to 90.5)	33.5 (27.1 to 39.9)	40.2 (33.8 to 46.5)	79.3 (70.8 to 87.8)
	NICE High-Risk	75.4 (67.5 to 83.3)	29.2 (22.9 to 35.5)	37.6 (31.3 to 43.8)	67.8 (58.0 to 77.6)
	Nice Moderate- and High-Risk	75.6 (70.3 to 80.9)	39.4 (27.6 to 51.2)	82.5 (77.6 to 87.5)	29.9 (20.3 to 39.5)
	qSOFA	75.4 (69.2 to 81.6)	31.6 (23.7 to 39.5)	60.3 (53.9 to 66.6)	48.3 (37.8 to 58.8)
	NEWS >4	79.5 (71.1 to 88.0)	30.3 (24.3 to 36.2)	30.6 (24.6 to 36.5)	79.3 (70.8 to 87.8)

Legend:

Analysis of Scoring Systems vs outcome measures in patients presenting to the EMU with suspected infection (n=316). PPV = Positive predictive value, NPV = negative predictive value. PPV, NPV, Sensitivity, and Specificity show percentage and 95% confidence intervals.

For peer review only

Table 3:

	NEWS >4	*qSOFA*	*NICE MR and HR*	*NICE High-Risk*
SIRS	0.2 (P=0.893)	21.3 (P<0.001)	25.7 (P<0.001)	6.63 (P=0.01)
NICE High-Risk	5.9* (P=0.034)	5.9 (P=0.015)	8.5 (P=0.004)	.
NICE MR and HR	18.5* (P<0.001)	1.27 (P=0.259)	.	.
qSOFA	19.3* (P<0.001)	.	.	.

Legend:

McNemar's analysis of each predictive tool compared against one another in determining the need for escalated care in patients with suspected infection (n=316).

Nice MR and HR – Combined analysis of NICE moderate- and high-risk criteria.

Values represent row-listed diagnostic tool performed better than column-listed diagnostic tool.

* represents column-listed diagnostic tool performed better than row-listed diagnostic tool.

A p-value of <0.005 is considered significant following Bonferroni correction for multiple analysis.

For peer review only

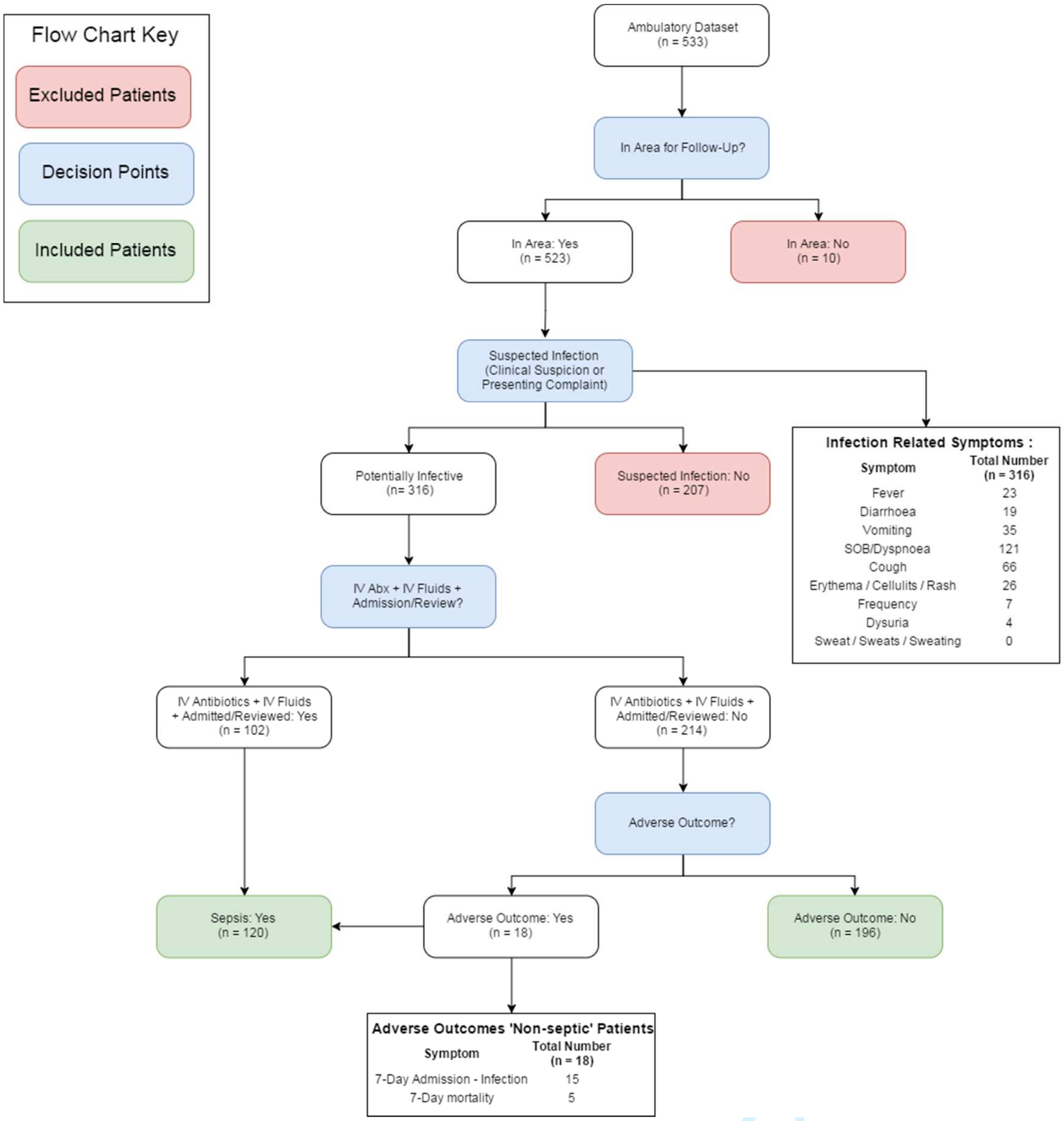

Figure 1 Patient flow from clinical presentation to outcomes

only

Supplementary Material

Supplementary Table 1:

Method	Score components: Available	Score components: Unavailable	Cut-off value
**NICE-HR** [1]			
	HR > 130 bpm	Mottled Skin	≥1
	RR > 25 min^{-1}	Cyanosis	
	New oxygen requirement	Non-blanching Rash	
	Systolic BP <90 mmHg	Urine Output	
	New Confusion	Systolic BP 40mmHG below normal	
**NICE-MR** [1]			
	HR 90 – 130 bpm	Steroid use / impaired immunity	≥1
	Systolic BP 90-100 mmHg	Trauma or Invasive Procedure	
	Reduced mobility or deterioration		
**qSOFA** [2]			
	GCS < 15	-	≥1
	RR > 21 min-1		
	Systolic BP ≤100 mmHg		
**SIRS** [3]			
	WCC <4.0 $x10^3$ or >12 x 10^3	-	≥2
	HR > 90 bpm		
	Temperature <36°C or >38°C		
	RR > 20 min^{-1}		
**NEWS** [4]			
	HR (multiple thresholds)	-	>4
	RR (multiple thresholds)		
	Oxygen Saturation (multiple thresholds)		
	Supplemental Oxygen (multiple thresholds)		
	Temperature (multiple thresholds)		
	Systolic BP (multiple thresholds)		
	Level of Consciousness (AVPU Scale)		

Legend

Components of diagnostic and severity assessment methods available and unavailable for use in this study. Cut-off values used as the threshold for predicting sepsis or poor patient outcome in this study. BPM – beats per minute, GCS – Glasgow Coma Scale, HR – Heart Rate, RR – Respiratory Rate, WCC – White Cell Count.

For peer review only

For peer review only - http://bmjopen.bmj.com/site/about/guidelines.xhtml

Supplementary Table 2:

Outcome Measure	*Test*	*PPV (%)*	*NPV (%)*	*Sensitivity (%)*	*Specificity (%)*
**Escalated care**					
	SIRS	55.6 (39.3 to 71.8)	80.8 (72.0 to 89.5)	57.1 (40.7 to 73.5)	79.7 (70.9 to 88.6)
	NICE High-Risk	40.4 (27.0 to 53.7)	77.4 (67.0 to 87.8)	60.0 (43.8 to 76.2)	60.8 (50.0 to 71.5)
	Nice Moderate- and High-Risk	31.0 (21.9 to 40.1)	71.4 (47.8 to 95.1)	88.6 (78.0 to 99.1)	12.7 (5.3 to 20.0)
	qSOFA	35.0 (24.5 to 45.5)	79.4 (65.8 to 93.0)	80.0 (66.7 to 93.3)	34.2 (23.7 to 44.6)
	NEWS >4	53.3 (35.5 to 71.2)	77.4 (68.4 to 86.3)	45.7 (29.2 to 62.2)	82.3 (73.9 to 90.7)
**30-Day Mortality**					
	SIRS	19.4 (6.5 to 32.4)	92.3 (86.4 to 98.2)	53.8 (26.7 to 80.9)	71.3 (62.5 to 80.1)
	NICE High-Risk	13.5 (4.2 to 22.7)	90.3 (83.0 to 97.7)	53.8 (26.7 to 80.9)	55.4 (45.8 to 65.1)
	Nice Moderate- and High-Risk	13.0 (6.4 to 19.6)	100.0 (100.0 to 100.0)	100.0 (100.0 to 100.0)	13.9 (7.1 to 20.6)
	qSOFA	13.8 (6.2 to 21.3)	94.1 (86.2 to 100.0)	84.6 (65.0 to 100.0)	31.7 (22.6 to 40.8)
	NEWS >4	20.0 (5.7 to 34.3)	91.7 (85.8 to 97.6)	46.2 (19.1 to 73.3)	76.2 (67.9 to 84.5)
**30-Day Admission**					
	SIRS	88.9 (78.6 to 99.2)	32.1 (21.7 to 42.4)	37.6 (27.3 to 47.9)	86.2 (73.7 to 98.8)
	NICE High-Risk	80.8 (70.1 to 91.5)	30.6 (19.2 to 42.1)	49.4 (38.8 to 60.0)	65.5 (48.2 to 82.8)
	Nice Moderate- and High-Risk	75.0 (66.5 to 83.5)	28.6 (4.9 to 52.2)	88.2 (81.4 to 95.1)	13.8 (1.2 to 26.3)
	qSOFA	77.5 (68.3 to 86.7)	32.4 (16.6 to 48.1)	72.9 (63.5 to 82.4)	37.9 (20.3 to 55.6)
	NEWS >4	93.3 (84.4 to 100.0)	32.1 (22.2 to 42.1)	32.9 (22.9 to 42.9)	93.1 (83.9 to 100.0)
**30-Day Composite**					
	SIRS	88.9 (78.6 to 99.2)	30.8 (20.5 to 41.0)	37.2 (27.0 to 47.4)	85.7 (72.8 to 98.7)
	NICE High-Risk	80.8 (70.1 to 91.5)	29.0 (17.7 to 40.3)	48.8 (38.3 to 59.4)	64.3 (46.5 to 82.0)
	Nice Moderate- and High-Risk	76.0 (67.6 to 84.4)	28.6 (4.9 to 52.2)	88.4 (81.6 to 95.1)	14.3 (1.3 to 27.2)
	qSOFA	77.5 (68.3 to 86.7)	29.4 (14.1 to 44.7)	72.1 (62.6 to 81.6)	35.7 (18.0 to 53.5)
	NEWS >4	93.3 (84.4 to 100.0)	31.0 (21.1 to 40.8)	32.6 (22.7 to 42.5)	92.9 (83.3 to 100.0)

For peer review only - http://bmjopen.bmj.com/site/about/guidelines.xhtml

Legend:

Analysis of Scoring Systems vs outcome measures in patients >85 years old with suspected infection (n=114). PPV = Positive predictive value, NPV = negative predictive value. PPV, NPV, Sensitivity, and Specificity show percentage and 95% confidence intervals.

For peer review only

For peer review only - http://bmjopen.bmj.com/site/about/guidelines.xhtml

BMJ Open

Sepsis recognition tools in acute ambulatory care: associations with process of care and clinical outcomes in a service evaluation

Journal:	*BMJ Open*
Manuscript ID	bmjopen-2017-020497.R1
Article Type:	Research
Date Submitted by the Author:	14-Feb-2018
Complete List of Authors:	Camm, Christian; University of Oxford, Radcliffe Department of Medicine<br>Hayward, Gail; University of Oxford, Nuffield Department of Primary Care Health Sciences<br>Elias, Tania; Oxford University Hospitals NHS Foundation Trust, Geratology<br>Bowen, Jordan; Oxford University Hospitals NHS Foundation Trust, Geratology<br>Hassanzadeh, Roya; Imperial College London, Public Health and Primary Care<br>Fanshawe, Thomas; University of Oxford, Nuffield Department of Primary Care Health Sciences<br>Pendlebury, Sarah; Stroke Prevention Reseacrh Unit, Nuffield Department of Clinical Neurosciences; NIHR Oxford Biomedical Research Centre<br>Lasserson, Daniel; University of Birmingham, Institute of Applied Health Research; University of Oxford, Nuffield Department of Medicine
**Primary Subject Heading**:	Health services research
Secondary Subject Heading:	Diagnostics, General practice / Family practice, Infectious diseases
Keywords:	EPIDEMIOLOGY, GENERAL MEDICINE (see Internal Medicine), GERIATRIC MEDICINE, Quality in health care < HEALTH SERVICES ADMINISTRATION & MANAGEMENT

SCHOLARONE™
Manuscripts

Sepsis recognition tools in acute ambulatory care: associations with process of care and clinical outcomes in a service evaluation

C Fielder Camm[1], Gail Hayward[2], Tania C N Elias[3,4], Jordan S T Bowen[3,4], Roya Hassanzadeh[3,5], Thomas Fanshawe[2], Sarah T Pendlebury,[4,6,7] Daniel S Lasserson[8]

[1]Radcliffe Department of Medicine, University of Oxford, Oxford, UK

[2]Nuffield Department of Primary Care Health Sciences, University of Oxford, Oxford, UK

[3]Emergency Multidisciplinary Unit, Abingdon Hospital, Oxford Health NHS Foundation Trust, Oxford, UK

[4]Department of Geratology, Oxford University Hospitals NHS Foundation Trust, Oxford, UK

[5]Department of Public Health and Primary Care, Imperial College London, London, UK

[6]Centre for Prevention of Stroke and Dementia, Nuffield Department of Clinical Neurosciences, University of Oxford, Oxford UK.

[7]NIHR Oxford Biomedical Research Centre, John Radcliffe Hospital Oxford, UK.

[8]Nuffield Department of Medicine, University of Oxford, Oxford, UK

[9]Institute of Applied Health Research, College of Medical and Dental Sciences, University of Birmingham, Birmingham, UK

Correspondence to:

Prof Daniel Lasserson, Professor of Ambulatory Care, Murray Learning Centre, Institute of Applied Health Research, College of Medical and Dental Sciences, University of Birmingham, Edgbaston, Birmingham, B15 2 TT.

D.S.Lasserson@bham.ac.uk

Manuscript Word Count: 3005 Words

For peer review only

Abstract

Objective

To assess the performance of currently available sepsis recognition tools in patients referred to a community based acute ambulatory care unit.

Design

Service evaluation of consecutive patients over a four month period.

Setting

Community based acute ambulatory care unit

Data collection and outcome measures:

Observations, blood results, and outcome data were analysed from patients with a suspected infection. Clinical features at first assessment were used to populate sepsis recognition tools including: systemic inflammatory responses syndrome (SIRS) criteria, national early warning score (NEWS), quick sequential organ failure assessment (qSOFA), and National Institute for Health and Care Excellence (NICE) criteria. Scores were assessed against the clinical need for escalated care (use of IV antibiotics, fluids, ongoing ambulatory care or hospital treatment) and poor clinical outcome (all-cause mortality and readmission at 30 days after index assessment).

Results

Of 533 patients (median age 81 years), 316 had suspected infection with 120 patients requiring care escalated beyond simple community care. SIRS had the highest positive predictive value (50.9%, 95% confidence interval (CI) [41.6% to 60.3%]) and negative predictive value (68.9%, 95% CI [62.6% to 75.3%]) for the need for escalated care. Both NEWS and SIRS were better at predicting the need for escalated care than qSOFA and NICE criteria in patients with suspected infection (all $p<0.001$). While new onset confusion predicted the need for escalated care for infection in patients ≥85 years old (n= 114), 23.7% of patients ≥85 years had new onset confusion without evidence for infection.

Conclusions

Acute ambulatory care clinicians should use caution in applying the new NICE endorsed criteria for determining need for intravenous therapy and hospital based location of care. NICE criteria have poorer performance when compared against NEWS and SIRS and new onset confusion was prevalent in patients aged ≥ 85 years without infection.

For peer review only

Strengths and limitations of this study

- Acute ambulatory care is applicable to an increasingly prevalent healthcare delivery model
- Consecutive patient evaluation to minimise selection bias
- Not all elements of the sepsis recognition models were available for scoring

Key Words

General Medicine, Geriatric Medicine, Quality in Healthcare, Epidemiology

For peer review only

Introduction

Sepsis, defined as 'life-threatening organ dysfunction caused by dysregulated host response to infection',[1] has a high mortality[2] but early recognition allows for prompt and effective treatment.[3] Therefore, tools for accurate recognition are needed at the earliest possible point after patients attend an initial healthcare assessment setting.

The Third International Consensus (SEPSIS-3) advocates the Sequential Organ Failure Assessment (SOFA)[1] score to evaluate sepsis. However, SOFA is not practical to use in the community and the Quick Sequential Organ Failure Assessment (qSOFA) score has been developed for use in rapid triage and in settings without access to blood tests.[4] The National Institute of Health and Care Excellence (NICE) has recently introduced new guidance regarding sepsis, including a tool for the diagnosis and triage of patients with suspected sepsis in community settings.[2] The use of the qSOFA, and the NICE high-risk and moderate risk criteria (NICE-HR and NICE-MR respectively) are a significant departure from the older systemic inflammatory response syndrome (SIRS) criteria.[5] In addition to sepsis recognition, the National Early Warning Score (NEWS) has been advocated by the Royal College of Physicians to track the clinical condition of patients presenting acutely for hospital and community assessment.[6]

New tools for sepsis recognition have not been evaluated in all healthcare environments that provide initial clinical assessments. Lower sensitivity or specificity of either qSOFA or NICE criteria compared with existing tools for detecting sepsis at initial assessment, particularly in the community, could result in poorer care overall. Lower sensitivity results in missed cases of sepsis compared with current recognition tools and lower specificity results in the unnecessary escalation of care including rapid transfer from community settings to emergency departments, unnecessary use of broad spectrum antibiotics and rapid fluid administration.

The performance of newer recognition scores, such as qSOFA and the NICE criteria, may vary with clinical setting. Low specificity of NICE criteria has been suggested by the large number of acute medical unit admissions it detects as needing rapid assessment within one hour, in comparison with qSOFA and SIRS.[7 8] Furthermore, among emergency department patients with low qSOFA scores, a significant proportion require critical care interventions which would not have been anticipated using qSOFA[9] and the now abandoned SIRS criteria have shown more accurate prediction of death than qSOFA in some emergency department cohorts.[10] Given that in intensive care unit patients, SOFA has a better performance than SIRS,[11 12] it is likely that the clinical setting and nature of the patient cohorts in which these tools are applied have a strong influence on their accuracy to detect sepsis and stratify patients based on risk of poor outcomes.

The acute ambulatory care model, which is recommended as a strategy in acute medicine to meet increasing demands for care within constrained resources,[13] represents a particular challenge for the recognition of sepsis. Whilst ambulatory care is becoming increasingly prevalent,[14] there are very few tools designed to support clinical decision making in the ambulatory care environment. Patients who are deemed suitable for ambulatory care do not have very disordered

For peer review only - http://bmjopen.bmj.com/site/about/guidelines.xhtml

physiology[15], even though there may be an emerging underlying significant disease process. Furthermore, identification of sepsis in older patients with frailty is complex as acute functional decline has many other causes,[16] yet mortality and morbidity related to sepsis is higher in the older population.[17] One of the critical decisions in ambulatory care management of suspected infection is selecting which patients can be safely discharged with oral therapy and follow up from their general practitioner and which patients are at higher risk of clinical deterioration and require care that is enhanced, either through an ambulatory platform with daily parenteral antibiotics with senior clinical review, or medical admission.

The ambulatory care clinician, faced with the diagnostic and management challenge of early sepsis recognition and the need to safely manage patients on an ambulatory pathway, can choose from four different sepsis recognition tools (SIRS, NEWS, NICE-HR/MR, and qSOFA). This study aims to evaluate the performance of these tools by examining their association with processes of care and clinical outcomes in patients with suspected infection in an acute ambulatory care setting. Given the uncertainty about application to older people in whom there is a wide differential diagnosis for acute frailty syndromes such as confusion, we undertook a subgroup analysis in patients 85 years or older.

Methods

The direct care team collected healthcare data as part of routinely provided healthcare from consecutive patients presenting to Abingdon Hospital's Emergency Multidisciplinary Unit (EMU) run by Oxford Health NHS Foundation Trust between 12/08/2015 and 08/12/2015. Patients were included if they were resident within Oxfordshire, allowing for appropriate follow-up details to be obtained, and had a clinical phenotype suggesting possible infection, in keeping with the recommended application of sepsis screening tools.[5] The use of routine healthcare data collected by the direct care team for the purposes of determining adherence to national guidance and application of clinical prediction tools to inform future service development was prospectively approved as a service evaluation by Oxford University Hospitals NHS Foundation Trust (providers of the medical care on EMU), with audit datix reference number 3812, and permission to publish was granted by Oxford Health NHS Foundation Trust R&D Department.

Study setting:

The EMU model of care provides assessment and treatment for adults with acute illness where an ambulatory treatment path is considered likely by the initial contact healthcare provider. Facilities include point of care blood testing (electrolytes, renal function, blood gases, lactate, C-reactive protein, troponin, INR), plain X Ray, and intravenous delivery of medications and fluids. Referrals are primarily from general practitioners and paramedic teams in the community. Patients whose care needs cannot be met with an ambulatory pathway are admitted to either a community hospital or an associated acute hospital.

For peer review only

Process of care and clinical outcomes:

Details regarding the patients' presentation, investigations, treatment, and outcome were prospectively documented at the time of assessment on the EMU. Patients' initial assessment was undertaken using a structured clinical clerking proforma including a brief cognitive test (the abbreviated mental test score (AMTS) and delirium screen) as described previously.[18] Data for analyses were extracted from the clerking proforma supplemented by information from ambulance sheets, GP referral letters, and communications with patients and relatives as listed in Supplementary Table 1. Patients were considered to have a possible infection if a clinical suspicion of infection was recorded by the treating clinician or if the presenting complaint suggested an infective cause for their symptoms. Processes of care included use of intravenous (IV) antibiotics, IV fluids and pathway of care (ambulatory vs hospital) and re-admission within 30 days. Clinical outcome was 30-day all-cause mortality.

We tested the ability of sepsis scoring systems to select patients who require continued monitoring and treatment from the medical team, either via ambulatory or bed based pathways. Patients were considered to have required escalated care for an infection if *all* the following conditions were met: (1) the patient had a possible infection, (2) the patient received IV antibiotics and IV fluids, (3) the patient was either admitted to an inpatient facility or subsequently brought back to the EMU for ≥1 daily reviews, or enrolled in the 'hospital @ home' programme to receive ongoing IV antibiotics and daily review after initial EMU assessment. Furthermore, patients with suspected infection who did not meet conditions (2) and (3) were considered to have needed escalated care if they died within 7-days of review or required re-admission for infection within 30 days. This strategy was used as it collectively identifies patients for whom initial discharge from ambulatory care with no or oral antibiotics may have been inappropriate.

Sepsis Scoring Systems:

We evaluated the accuracy of SIRS,[18] qSOFA,[6] NEWS,[2] NICE-HR and NICE-MR from NICE Guideline NG51[7] for detecting the need for escalated care to treat suspected bacterial infection. Each method was undertaken in accordance with guidance provided by issuing bodies. Features associated with these tools available in our data-set and cut-off values for prediction of sepsis or poor outcome are detailed in Supplementary Table 2. Within selected scoring systems, (NICE-MR, NICE-HR), some variables are not systematically available at the first assessment in an acute setting (e.g. urine output, usual blood pressure values for calculation of difference from observed value) and Supplementary Table 2 lists these variables together with any additional clinical features that were not systematically sought within the clerking proforma. The scores were calculated without use of these items which would reflect real world practice after implementation. A cut-off score of >4 was used for NEWS in line with guidance from the Royal College of Physicians (London) which details this threshold for separating low-risk patients from those at increased risk.[7] For cases in which individual items required for a score were

missing, the score for that individual was calculated without that item, in line with clinical practice. During the analysis NICE-MR and HR were combined in some analyses to replicate the use of the moderate criteria in clinical practice. We did not separately analyse the Sepsis Trust Red/Amber flag system (https://sepsistrust.org/education/clinical-tools/) as features of this system were incorporated into the new NICE guidelines.

Statistical Analysis:

Continuous variables are expressed as either mean (standard deviation) or median (interquartile range [IQR]) and compared across suspected infection/no suspected infection and escalated care/no escalated care groups using the independent student's T-test or Mann-Whitney U-test respectively. Categorical and ordinal variables are reported as frequency (percentage) and compared between groups using the χ^2 or Fisher's exact test. The diagnostic accuracy of each predictive tool was assessed using positive predictive value (PPV), negative predictive value (NPV), sensitivity and specificity, with 95% confidence intervals. Predictive tools were compared with one another using McNemar's test, and we consider p-values of less than 0.005 to be statistically significant in these comparisons (based on a conservative Bonferroni correction for ten comparisons). The performance of sepsis tools was assessed in the whole dataset and in a subgroup of the oldest patients (≥85 years) in predicting the need for escalated care, mortality, and readmission within 30 days of index assessment. Analysis was performed using Version 9 of the SAS system for Windows. Copyright © 2002-2010 by SAS Institute Inc., Cary, NC, USA.

Results

A total of 533 patients were assessed during the observation time period. Ten patients were not local residents and did not have follow-up data and were excluded from the analysis (Figure 1). Demographic data of the study population are provided in Table 1. 316 patients were considered to have a potential infection with 120 patients requiring escalated treatment for infection. Features most significantly associated with need for escalated care were increased heart rate (89.6 (18.9) vs. 83.8 (17.8), p=0.006), increased temperature (37.0 (0.9) vs 36.6 (0.6), p<0.001), and increased white cell count (14.5 (17.4) vs. 9.3 (3.7), p=0.003). In the whole cohort, there was no association between new onset confusion and need for escalated care (22/120 (20.0%) vs. 27/196 (14.0%), p=0.172). A full list of features associated with escalated care is displayed in Table 1.

Of 186 patients ≥85 years old (45% of the cohort), 114 patients had a suspected infection as the cause for their presentation. Among patients ≥85 years with suspected infection, those who met criteria for escalated care (n=35, 30.7%) also demonstrated the positive findings for the whole cohort in terms of difference in physiological markers, but new confusion was significantly associated with escalated care in this subgroup (Table 1).

The PPV, NPV, sensitivity and specificity of each assessment tool against the need for escalated care, 30-day mortality, 30-day admission, and a composite marker of 30-day mortality or admission for the cohort with suspected infection are

For peer review only - http://bmjopen.bmj.com/site/about/guidelines.xhtml

shown in Table 2. SIRS had the highest PPV (50.9%, 95% CI [41.6% to 60.3%]) and NPV (68.9%, 95% CI [62.6% to 75.3%]). Using McNemar's test, the combined high- and moderate-risk NICE criteria were shown to be significantly worse at predicting escalated care compared with SIRS ($p<0.001$), NEWS ($p<0.001$), and NICE High-risk criteria alone ($p=0.004$) (Table 3), a result driven by very low specificity of the combined high- and moderate-risk NICE criteria (21.4%, 95% CI [15.7 to 27.2]). Furthermore, both SIRS and NEWS were shown to have better performance against qSOFA (both $p<0.001$). NEWS and SIRS had the highest specificity for detecting 30-day mortality (67.0 95% CI [61.6 to 72.4] and 73.5 95% CI [68.5 to 78.6] respectively).

Among patients ≥85 years old, the PPV and NPV of both SIRS and NEWS were higher than NICE or qSOFA scores for escalated care and for the combined 30 day clinical outcome (Supplementary Table 3). Whilst NICE criteria had the highest sensitivity at detecting poor clinical outcomes (mortality or admission within 30 days) (88.4%), this was at the cost of very low specificity (14.3%). Receiver operator characteristic curves for the ability of the different sepsis scoring systems to identify patients requiring escalated care are shown in Supplementary Figure 1.

Discussion

Of the tools assessed, SIRS and NEWS were shown to have the highest PPV and NPV for escalated care in the dataset overall and in the subgroup of patients ≥ 85 years. Whilst NICE criteria and qSOFA had better performance in detecting requirement for escalated care in patients ≥85 years, their poorer specificity contributed to a lower PPV. The presence of new confusion was associated with escalated care among patients with suspected infection in the subgroup of patients aged ≥ 85 years.

Although SIRS was shown to be the most predictive of escalated care in patients with suspected infection in this study, the combined high and moderate risk NICE criteria would capture the most patients requiring escalated care but at the cost of very low specificity i.e. a high false positive rate. These results contrast with the previously suggested limitations in predictive ability for poor outcomes when compared against other potential sepsis recognition and stratification tools which have led to a move away from using SIRS.[5] This may reflect applicability in different patient cohorts as Kaukonen *et al.* demonstrated that 12.1% of intensive care unit patients with severe sepsis did not meet ≥2 SIRS criteria.[2 19 20] However, they also showed that those with SIRS positive sepsis had significantly increased mortality compared with those not meeting these criteria. Churpek *et al.* demonstrated that 47% of an acute patient cohort met ≥2 SIRS criteria during admission and concluded that screening patients for sepsis based on these criteria would be unwise.[19] However, this paper did not select patients in whom infection was suspected and instead applied it to all admissions, irrespective of presenting complaint. A recent analysis from the same group has also suggested that in those admitted to hospital and suspected of

For peer review only

infection, discrimination for in-hospital mortality was highest using NEWS.[21] Our results add to this information by providing support for the use of NEWS in identifying patients at need of care escalation in a community setting.

The importance of identifying sepsis in the initial stages of clinical contact to allow for prompt and effective treatment has been well documented.[20] However, our results have shown that those systems endorsed by both NICE and Sepsis-3 have limitations related to high false-positive rates as compared with both SIRS and NEWS, which could lead to substantial over-treatment.

The introduction of new-onset confusion as a predictor of sepsis in NICE guidance risks increasing the use of inappropriate antibiotics in the older patient with frailty. Although our results show a significant association between the development of new confusion and the need for escalated care among patients ≥ 85 years with infection, there was a high prevalence of new onset confusion in this age group without any evidence of underlying infection. While infection is a principle cause of delirium,[3] there is limited high-quality evidence that altered mental state in older adults is predictive of sepsis itself [22] and delirium has a wide range of potential causes in older people.[5]

Our data show that the new combined NICE high-risk and moderate-risk criteria are likely to significantly increase the number of patients that are determined as potentially having sepsis at initial assessment in ambulatory care. As such, this is likely to increase the number of patients receiving intravenous antibiotics which may conflict with the national and global priorities of antibiotic stewardship.[22] Furthermore, in the ambulatory care setting their introduction may increase conversion to formal hospital admission which our data suggest will not be justified based on the clinical outcomes we recorded.

Limitations

Several limitations of this work are acknowledged. Data collection for this work was undertaken at a time when SIRS was the recommended assessment tool for potential sepsis. As such, incorporation bias may explain some degree of the correlation between this method of assessment and the diagnosis and treatment of sepsis. However, SIRS was not calculated as part of the standard assessment proforma in this cohort and white cell count was not available at initial review as it is not part of the point of care testing portfolio. This limitation would not explain the correlation between SIRS and NEWS with mortality at 30-days.

Not all elements of the new NICE criteria for high- and moderate-risk sepsis were available for this analysis. However, the limited specificity of both these methods in determining sepsis would only be worsened by the addition of further criteria to this analysis. Furthermore, we argue that several of those unavailable elements (e.g. Mottled Skin, Cyanosis, and Urine Output) represent those that would either not be available in the acute ambulatory setting or are based on subjective assessment and therefore more likely to vary between clinicians.

For peer review only

Conclusions:

This study demonstrates that new NICE recommended sepsis tools do not outperform the existing tools in an ambulatory care setting in predicting the need for escalated care for infection and clinical outcomes at 30 days. Although NICE tools had higher sensitivity, this was at the expense of very low specificity. SIRS had the highest predictive values but the requirement of a white cell count implies that it can only be used widely if this is tested at the point of care. Further research should be undertaken to determine applicability of NICE sepsis guidance in different healthcare settings where acutely unwell patients are assessed before widespread adoption in the NHS.

For peer review only

Funding Statement

CFC is funded by an NIHR Academic Clinical Fellowship. The NIHR Oxford Biomedical Research Centre supported DSL and SP with grant number AC14/035 supporting JSTB and TCNE. JSTB is also supported by the NIHR Oxford Collaboration for Leadership in Applied Health Research and Care (CLAHRC) This article presents independent research funded by the National Institute for Health Research (NIHR) Diagnostic Evidence Co-operative Oxford. The opinions expressed in this study are those of the authors and not necessarily those of the NIHR, the Department of Health or the NHS.

Contribution Statement

CFC undertook analyses and drafted the initial manuscript. GH and TF advised on analytical methodology, interpreted data and critically revised the manuscript. JSTB, TCNE and RH designed data collection methods, collected data, clinically interpreted analyses and critically revised the manuscript. SP designed the service evaluation, data collection methods and critically revised the manuscript. DSL designed the service evaluation, advised on analytical methods, interpreted data and critically revised the manuscript.

Data sharing statement

No additional data are available

Conflict of Interest

None declared.

For peer review only

References

1. Fleischmann C, Scherag A, Adhikari NK, et al. Assessment of Global Incidence and Mortality of Hospital-treated Sepsis. Current Estimates and Limitations. Am J Respir Crit Care Med 2016;**193**(3):259-72.

2. Singer M, Deutschman CS, Seymour CW, et al. The Third International Consensus Definitions for Sepsis and Septic Shock (Sepsis-3). JAMA 2016;**315**(8):801-10.

3. Ferrer R, Martin-Loeches I, Phillips G, et al. Empiric antibiotic treatment reduces mortality in severe sepsis and septic shock from the first hour: results from a guideline-based performance improvement program. Crit Care Med 2014;**42**(8):1749-55.

4. Vincent JL, Moreno R, Takala J, et al. The SOFA (Sepsis-related Organ Failure Assessment) score to describe organ dysfunction/failure. On behalf of the Working Group on Sepsis-Related Problems of the European Society of Intensive Care Medicine. Intensive Care Med 1996;**22**(7):707-10.

5. National Institute of Health and Care Excellence. NICE Guideline 51 - Sepsis: recognition, assessment and early management: National Institute of Health and Care Excellence, 2016.

6. Bone RC, Balk RA, Cerra FB, et al. Definitions for Sepsis and Organ Failure and Guidelines for the Use of Innovative Therapies in Sepsis. Chest 1992;**101**(6):1644-55.

7. National Early Warning Score (NEWS). Standardising the assessment of acute-illness severity in the NHS: Royal College of Physicians (London), 2012.

8. Smith GB, Prytherch DR, Schmidt PE, et al. Review and performance evaluation of aggregate weighted 'track and trigger' systems. Resuscitation 2008;**77**(2):170-9.

9. Thompson A, Stevens M, Collin I, et al. Evolving sepsis definitions and their impact on Acute Medical Units. Acute medicine 2017;**16**(1):25-29.

10. Moskowitz A, Patel PV, Grossestreuer AV, et al. Quick Sequential Organ Failure Assessment and Systemic Inflammatory Response Syndrome Criteria as Predictors of Critical Care Intervention Among Patients With Suspected Infection. Crit Care Med 2017.

11. Askim A, Moser F, Gustad LT, et al. Poor performance of quick-SOFA (qSOFA) score in predicting severe sepsis and mortality - a prospective study of patients admitted with infection to the emergency department. Scandinavian journal of trauma, resuscitation and emergency medicine 2017;**25**(1):56.

12. Williams JM, Greenslade JH, McKenzie JV, et al. Systemic Inflammatory Response Syndrome, Quick Sequential Organ Function Assessment, and Organ Dysfunction: Insights From a Prospective Database of ED Patients With Infection. Chest 2017;**151**(3):586-96.

13. Commission FH. Future hospital: caring for medical patients. A report from the Future Hospital Commission to the Royal College of Physicians. London, 2013.

14. Nasa P, Juneja D, Singh O, et al. Severe sepsis and its impact on outcome in elderly and very elderly patients admitted in intensive care unit. J Intensive Care Med 2012;**27**(3):179-83.

15. Raith EP, Udy AA, Bailey M, et al. Prognostic Accuracy of the SOFA Score, SIRS Criteria, and qSOFA Score for In-Hospital Mortality Among Adults With Suspected Infection Admitted to the Intensive Care Unit. Jama 2017;**317**(3):290-300.

16. Ala L, Mack J, Shaw R, et al. Selecting ambulatory emergency care (AEC) patients from the medical emergency in-take: the derivation and validation of the Amb score. Clinical medicine (London, England) 2012;**12**(5):420-6.

17. Girard TD, Opal SM, Ely EW. Insights into severe sepsis in older patients: from epidemiology to evidence-based management. Clin Infect Dis 2005;**40**(5):719-27.

18. Pendlebury ST, Lovett NG, Smith SC, et al. Observational, longitudinal study of delirium in consecutive unselected acute medical admissions: age-specific rates and associated factors, mortality and re-admission. BMJ Open 2015;**5**(11):e007808.

For peer review only

19. Kaukonen KM, Bailey M, Pilcher D, et al. Systemic inflammatory response syndrome criteria in defining severe sepsis. N Engl J Med 2015;**372**(17):1629-38.

20. Churpek MM, Zadravecz FJ, Winslow C, et al. Incidence and Prognostic Value of the Systemic Inflammatory Response Syndrome and Organ Dysfunctions in Ward Patients. Am J Respir Crit Care Med 2015;**192**(8):958-64.

21. Churpek MM, Snyder A, Han X, et al. Quick Sepsis-related Organ Failure Assessment, Systemic Inflammatory Response Syndrome, and Early Warning Scores for Detecting Clinical Deterioration in Infected Patients outside the Intensive Care Unit. Am J Respir Crit Care Med 2017;**195**(7):906-11.

22. Fong TG, Tulebaev SR, Inouye SK. Delirium in elderly adults: diagnosis, prevention and treatment. Nat Rev Neurol 2009;**5**(4):210-20.

For peer review only

Figure 1 Patient flow from clinical presentation to outcomes

Tables

Table 1:

Item	All patients with suspected infection				≥85 years old				<85 years old			
		Need for escalated care				Need for escalated care				Need for escalated care		
	N	Yes	No	p-value	N	Yes	No	p-value	N	Yes	No	p-value
Subjects	316	120 (38.0%)	196 (62.0%)	.	114	35 (30.7%)	79 (69.3%)	.	202	85 (42.1%)	117 (57.9%)	.
Proportion Male	316	57 (47.5%)	79 (40.3%)	0.210	114	10 (28.6%)	33 (41.8%)	0.180	202	47 (55.3%)	46 (39.3%)	0.024
Age (years)	316	74.5 (16.6)	75.2 (18.3)	0.708	114	90.2 (3.3)	89.7 (3.9)	0.442	202	68.0 (15.5)	65.4 (17.7)	0.283
Heart Rate (beats min^{-1})	315	89.6 (18.9)	83.8 (17.8)	0.006	114	89.5 (19.2)	79.29 (15.5)	0.003	201	89.6 (18.9)	86.9 (18.6)	0.306
Respiratory Rate	314	21.6 (6.4)	21.3 (5.3)	0.643	113	24.4 (8.2)	20.7 (5.2)	0.016	201	20.5 (5.1)	21.7 (5.4)	0.094
Systolic Blood Pressure	314	132.2 (20.1)	135.9 (26.7)	0.172	113	129.1 (21.4)	132.0 (26.1)	0.534	201	133.5 (19.5)	138.5 (26.9)	0.133
Oxygen Saturations (%)	313	96 (94 - 98)	97 (95 - 98)	0.007	113	96 (93 - 97)	97 (95 - 98)	0.009	200	96 (94 - 98)	97 (94 - 98)	0.132
Temperature (°C)	314	37.0 (0.9)	36.6 (0.6)	<0.001	113	36.9 (0.9)	36.5 (0.6)	0.044	201	37.1 (0.9)	36.7 (0.6)	0.001
AMTS	303	10 (8 - 10)	10 (9 - 10)	0.155	107	7 (5 - 9)	9 (7 - 10)	0.008	196	10 (9 - 10)	10 (9 - 10)	0.532
Known Dementia Diagnosis	316	21 (17.5%)	23 (11.7%)	0.151	114	9 (25.7%)	14 (17.7%)	0.327	202	12 (14.1%)	9 (7.7%)	0.140
New Confusion	316	22 (18.3%)	27 (13.8%)	0.172	114	15 (42.9%)	18 (22.8%)	0.012	202	7 (8.2%)	9 (7.7%)	0.769
White Cell Count(x 10^9/L)	274	14.5 (17.4)	9.3 (3.7)	0.003	99	13.9 (6.4)	9.3 (4.1)	0.001	175	14.7 (20.4)	9.3 (3.3)	0.028
Mortality (30-Day)	316	14 (11.7%)	11 (5.6%)	0.053	114	8 (22.9%)	5 (6.3%)	0.010	202	6 (7.1%)	6 (5.1%)	0.567
Barthel Score ≥15	316	80 (66.7%)	150 (76.5%)	0.056	114	17 (48.6%)	53 (67.1%)	0.061	202	63 (74.1%)	97 (82.9%)	0.129

Sepsis scores at initial presentation									
NEWS	316	3 (1 – 5)	3 (1 – 4)	114	4 (3 – 9)	3 (1 – 4)	202	3 (1 – 5)	3 (1 – 5)
SIRS criteria	316	1 (1 – 2)	1 (0 – 2)	114	2 (1 – 2)	1 (0 – 1)	202	1 (0 – 2)	1 (0 – 2)
qSOFA criteria	316	1 (0 – 1)	1 (0 – 1)	114	1 (1 – 2)	1 (0 – 1)	202	0 (0 – 1)	1 (0 – 1)
NICE High-Risk	316	45 (37.5%)	69 (35.2%)	114	21 (60.0%)	31 (39.2%)	202	24 (28.2%)	38 (32.5%)
Nice Moderate- and High-Risk	316	96 (80.0%)	154 (78.6%)	114	31 (88.6%)	69 (87.3%)	202	65 (76.5%)	85 (72.7%)

Legend:

Demographic characteristics of patients assessed on the EMU during this analysis with suspected infection (n=316). AMTS – Abbreviated Mental Test Score. Results displayed as N(%), mean (standard deviation), or median(inter-quartile range) unless otherwise indicated. For the scoring systems evaluated, all data points evaluated were available in the following numbers: SIRS (n=272), NICE High-Risk (n=314), NICE Moderate- and High-Risk (n=314), qSOFA (n=314), NEWS (n=313).

For peer review only

Table 2

Outcome Measure	*Test*	*PPV (%)*	*NPV (%)*	*Sensitivity (%)*	*Specificity (%)*
**Escalated care**					
	SIRS	50.9 (41.6 to 60.3)	68.9 (62.6 to 75.3)	46.7 (37.7 to 55.6)	72.4 (66.2 to 78.7)
	NICE High-Risk	39.5 (30.5 to 48.4)	62.9 (56.2 to 69.5)	37.5 (28.8 to 46.2)	64.8 (58.1 to 71.5)
	Nice Moderate- and High-Risk	38.4 (32.4 to 44.4)	63.6 (52.0 to 75.2)	80.0 (72.8 to 87.2)	21.4 (15.7 to 27.2)
	qSOFA	37.2 (30.2 to 44.2)	60.9 (52.6 to 69.2)	56.7 (47.8 to 65.5)	41.3 (34.4 to 48.2)
	NEWS >4	48.9 (38.4 to 59.3)	66.2 (60.1 to 72.4)	35.8 (27.3 to 44.4)	77.0 (71.2 to 82.9)
**30-Day Mortality**					
	SIRS	12.7 (6.5 to 19.0)	94.7 (91.6 to 97.7)	56.0 (36.5 to 75.5)	67.0 (61.6 to 72.4)
	NICE High-Risk	12.3 (6.3 to 18.3)	94.6 (91.4 to 97.7)	56.0 (36.5 to 75.5)	65.6 (60.2 to 71.1)
	Nice Moderate- and High-Risk	9.6 (5.9 to 13.3)	98.5 (95.5 to 100.0)	96.0 (88.3 to 100.0)	22.3 (17.6 to 27.1)
	qSOFA	11.5 (6.9 to 16.1)	97.0 (94.1 to 99.9)	84.0 (69.6 to 98.4)	44.3 (38.6 to 50.0)
	NEWS >4	12.5 (5.6 to 19.4)	93.9 (90.7 to 97.0)	44.0 (24.5 to 63.5)	73.5 (68.5 to 78.6)
**30-Day readmission**					
	SIRS	82.7 (75.7 to 89.8)	34.0 (27.5 to 40.4)	40.1 (33.7 to 46.5)	78.7 (70.1 to 87.2)
	NICE High-Risk	74.6 (66.6 to 82.6)	29.7 (23.4 to 36.0)	37.4 (31.1 to 43.7)	67.4 (57.7 to 77.2)
	Nice Moderate- and High-Risk	74.8 (69.4 to 80.2)	39.4 (27.6 to 51.2)	82.4 (77.4 to 87.3)	29.2 (19.8 to 38.7)
	qSOFA	74.9 (68.6 to 81.1)	32.3 (24.4 to 40.3)	60.4 (54.0 to 66.7)	48.3 (37.9 to 58.7)
	NEWS >4	78.4 (69.8 to 87.0)	30.7 (24.7 to 36.7)	30.4 (24.4 to 36.4)	78.7 (70.1 to 87.2)
**30-Day Composite**					
	SIRS	83.6 (76.7 to 90.5)	33.5 (27.1 to 39.9)	40.2 (33.8 to 46.5)	79.3 (70.8 to 87.8)
	NICE High-Risk	75.4 (67.5 to 83.3)	29.2 (22.9 to 35.5)	37.6 (31.3 to 43.8)	67.8 (58.0 to 77.6)
	Nice Moderate- and High-Risk	75.6 (70.3 to 80.9)	39.4 (27.6 to 51.2)	82.5 (77.6 to 87.5)	29.9 (20.3 to 39.5)
	qSOFA	75.4 (69.2 to 81.6)	31.6 (23.7 to 39.5)	60.3 (53.9 to 66.6)	48.3 (37.8 to 58.8)
	NEWS >4	79.5 (71.1 to 88.0)	30.3 (24.3 to 36.2)	30.6 (24.6 to 36.5)	79.3 (70.8 to 87.8)

Legend:

Analysis of Scoring Systems vs outcome measures in patients presenting to the EMU with suspected infection (n=316). PPV = Positive predictive value, NPV = negative predictive value. PPV, NPV, Sensitivity, and Specificity show percentage and 95% confidence intervals.

For peer review only

Table 3:

	NEWS >4	*qSOFA*	*NICE MR and HR*	*NICE High-Risk*
SIRS	0.2 (P=0.893)	21.3 (P<0.001)	25.7 (P<0.001)	6.63 (P=0.01)
NICE High-Risk	5.9* (P=0.034)	5.9 (P=0.015)	8.5 (P=0.004)	.
NICE MR and HR	18.5* (P<0.001)	1.27 (P=0.259)	.	.
qSOFA	19.3* (P<0.001)	.	.	.

Legend:

McNemar's analysis of each predictive tool compared against one another in determining the need for escalated care in patients with suspected infection (n=316).

Nice MR and HR – Combined analysis of NICE moderate- and high-risk criteria.

Values represent row-listed diagnostic tool performed better than column-listed diagnostic tool.

* represents column-listed diagnostic tool performed better than row-listed diagnostic tool.

A p-value of <0.005 is considered significant following Bonferroni correction for multiple analysis.

For peer review only

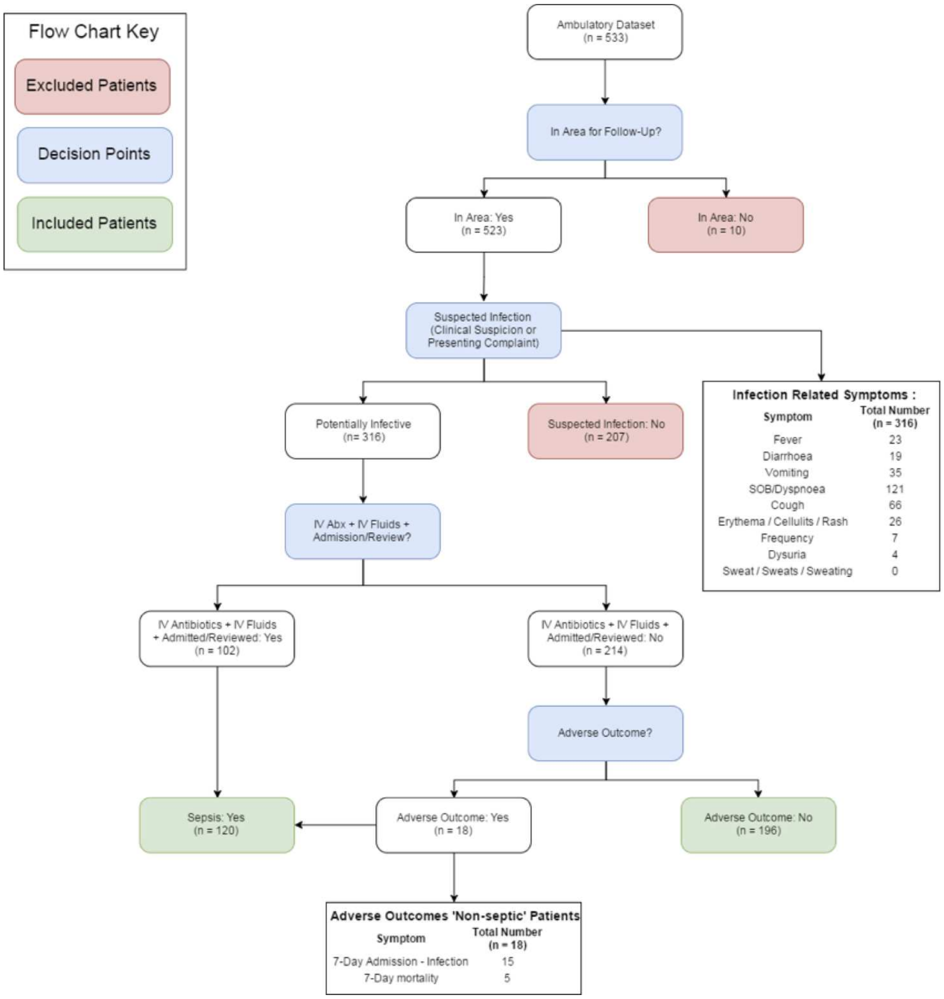

Patient flow from clinical presentation to outcomes

318x314mm (300 x 300 DPI)

Supplementary Material

Supplementary Table 1:

Data Field		*Data type*
Demographics		
	Study ID	Numerical
	Age	Numerical
	Sex	Categorical
	Weight	Numerical
	Height	Numerical
Initial Attendance Information		
	Date of first attendance	Date
	Attendance time	Time
	Was the patient accompanied	Boolean
	Most senior clinician reviewing patient on initial attendance	Categorical
Patient Background		
	Comorbidities	Free Text
	Known Dementia	Boolean
Presenting complaints		
	Presenting Complaint	Free Text
	Confusion	Boolean
	Delirium	Boolean
	SOB	Boolean
	Confusion / altered behaviour	Boolean
	Decreased mobility	Boolean
	Increased care needs	Boolean
	Falls	Boolean
	Chest Pain, tachycardia, or palpitations	Boolean
	Other respiratory (cough, wheeze, sore throat)	Boolean
	Abdominal symptoms (pain, bloating, diarrhoea, nausea, vomiting, constipation	Boolean
	Urinary or GU symptoms (dysuria, catheter problems, retention)	Boolean
	Fatigue, weight loss, reduced oral intake	Boolean
	Bilateral leg swelling	Boolean
	Pain (back, hip/groin, shoulder, knee, wrist, toe, facial)	Boolean
	Biochemical or haematological derangement (electrolytes, renal function, LFTs)	Boolean
	Symptomatic anaemia	Boolean
	Glycaemic derangement	Boolean
	Other neurological (seizure, headache, tremor, visual symptoms, virtigo, tinnitus)	Boolean
	PR bleed / malaena	Boolean
	Leg weakness	Boolean
	Fever	Boolean
	Rash / erythema (incl face)	Boolean
	Unilateral swelling	Boolean
	Collapse	Boolean
	Drowsiness	Boolean
	Dizziness / unsteadiness	Boolean
	Anxiety / low mood	Boolean
Observations on Admission		
	Heart rate	Numerical
	Systolic blood pressure	Numerical
	Diastolic blood pressure	Numerical
	Oxygen saturations	Numerical

For peer review only

Respiratory rate	Numerical
Temperature	Numerical
Postural drop in blood pressure	Boolean
AMTS	Ordinal
Glasgow coma score	Ordinal
**EMU Blood Results (categories)**	
Full blood count panel	Numerical
Urea and electrolytes panel	Numerical
C-reactive protein	Numerical
Liver function panel	Numerical
INR	Numerical
Lactate	Numerical
Troponin	Numerical
**Initial Diagnosis**	
Initial Diagnosis	Free text
Suspected infection	Boolean
Infection Source	Free text
**Treatments initiated**	
IV fluids	Boolean
Blood transfusion	Boolean
Nebulisers	Boolean
IV antibiotics	Boolean
Oral antibiotics	Boolean
IV iron	Boolean
IV furosemide	Boolean
Medications held	Boolean
Laxatives	Boolean
Analgesia	Boolean
Management	Free Text
**Outcomes**	
Initial plan (ambulation or admission)	Categorical
Escalation unit	Categorical
Documented reason for escalation	Free text
Date of death	Date
Discharge Date	Date
Number of visits to EMU	Numerical
Length of stay (if admitted)	Numerical
Date of EMU review	Date
Reason for EMU review	Free text
Unexpected re-admission within 30 days	Boolean

Legend:

Relevant variables collected as part of routine healthcare provision

For peer review only

Supplementary Table 2

	Score components		
Method	*Available*	*Unavailable*	*Cut-off value*
**NICE-HR** [1]			
	HR > 130 bpm	Mottled Skin	≥1
	RR > 25 min^{-1}	Cyanosis	
	New oxygen requirement	Non-blanching Rash	
	Systolic BP <90 mmHg	Urine Output	
	New Confusion	Systolic BP 40mmHG below normal	
**NICE-MR** [1]			
	HR 90 – 130 bpm	Steroid use / impaired immunity	≥1
	RR 21-24 min^{-1}	Trauma or Invasive Procedure	
	Systolic BP 90-100 mmHg	Redness/swelling/discharge at surgical site or breakdown of wound	
	Temperature <36°C		
	Reduced mobility or deterioration		
**qSOFA** [2]			
	GCS < 15	-	≥1
	RR > 21 min-1		
	Systolic BP ≤100 mmHg		
**SIRS** [3]			
	WCC <4.0 $x10^3$ or >12 x 10^3	-	≥2
	HR > 90 bpm		
	Temperature <36°C or >38°C		
	RR > 20 min^{-1}		
**NEWS** [4]			
	HR (multiple thresholds)	-	>4
	RR (multiple thresholds)		
	Oxygen Saturation (multiple thresholds)		
	Supplemental Oxygen (multiple thresholds)		
	Temperature (multiple thresholds)		
	Systolic BP (multiple thresholds)		
	Level of Consciousness (AVPU Scale)		

Legend

Components of diagnostic and severity assessment methods available and unavailable for use in this study. Cut-off values used as the threshold for predicting sepsis or poor patient outcome in this study. BPM – beats per minute, GCS – Glasgow Coma Scale, HR – Heart Rate, RR – Respiratory Rate, WCC – White Cell Count.

For peer review only

For peer review only - http://bmjopen.bmj.com/site/about/guidelines.xhtml

Supplementary Table 3:

Outcome Measure	*Test*	*PPV (%)*	*NPV (%)*	*Sensitivity (%)*	*Specificity (%)*
**Escalated care**					
	SIRS	55.6 (39.3 to 71.8)	80.8 (72.0 to 89.5)	57.1 (40.7 to 73.5)	79.7 (70.9 to 88.6)
	NICE High-Risk	40.4 (27.0 to 53.7)	77.4 (67.0 to 87.8)	60.0 (43.8 to 76.2)	60.8 (50.0 to 71.5)
	Nice Moderate- and High-Risk	31.0 (21.9 to 40.1)	71.4 (47.8 to 95.1)	88.6 (78.0 to 99.1)	12.7 (5.3 to 20.0)
	qSOFA	35.0 (24.5 to 45.5)	79.4 (65.8 to 93.0)	80.0 (66.7 to 93.3)	34.2 (23.7 to 44.6)
	NEWS >4	53.3 (35.5 to 71.2)	77.4 (68.4 to 86.3)	45.7 (29.2 to 62.2)	82.3 (73.9 to 90.7)
**30-Day Mortality**					
	SIRS	19.4 (6.5 to 32.4)	92.3 (86.4 to 98.2)	53.8 (26.7 to 80.9)	71.3 (62.5 to 80.1)
	NICE High-Risk	13.5 (4.2 to 22.7)	90.3 (83.0 to 97.7)	53.8 (26.7 to 80.9)	55.4 (45.8 to 65.1)
	Nice Moderate- and High-Risk	13.0 (6.4 to 19.6)	100.0 (100.0 to 100.0)	100.0 (100.0 to 100.0)	13.9 (7.1 to 20.6)
	qSOFA	13.8 (6.2 to 21.3)	94.1 (86.2 to 100.0)	84.6 (65.0 to 100.0)	31.7 (22.6 to 40.8)
	NEWS >4	20.0 (5.7 to 34.3)	91.7 (85.8 to 97.6)	46.2 (19.1 to 73.3)	76.2 (67.9 to 84.5)
**30-Day Admission**					
	SIRS	88.9 (78.6 to 99.2)	32.1 (21.7 to 42.4)	37.6 (27.3 to 47.9)	86.2 (73.7 to 98.8)
	NICE High-Risk	80.8 (70.1 to 91.5)	30.6 (19.2 to 42.1)	49.4 (38.8 to 60.0)	65.5 (48.2 to 82.8)
	Nice Moderate- and High-Risk	75.0 (66.5 to 83.5)	28.6 (4.9 to 52.2)	88.2 (81.4 to 95.1)	13.8 (1.2 to 26.3)
	qSOFA	77.5 (68.3 to 86.7)	32.4 (16.6 to 48.1)	72.9 (63.5 to 82.4)	37.9 (20.3 to 55.6)
	NEWS >4	93.3 (84.4 to 100.0)	32.1 (22.2 to 42.1)	32.9 (22.9 to 42.9)	93.1 (83.9 to 100.0)
**30-Day Composite**					
	SIRS	88.9 (78.6 to 99.2)	30.8 (20.5 to 41.0)	37.2 (27.0 to 47.4)	85.7 (72.8 to 98.7)
	NICE High-Risk	80.8 (70.1 to 91.5)	29.0 (17.7 to 40.3)	48.8 (38.3 to 59.4)	64.3 (46.5 to 82.0)
	Nice Moderate- and High-Risk	76.0 (67.6 to 84.4)	28.6 (4.9 to 52.2)	88.4 (81.6 to 95.1)	14.3 (1.3 to 27.2)
	qSOFA	77.5 (68.3 to 86.7)	29.4 (14.1 to 44.7)	72.1 (62.6 to 81.6)	35.7 (18.0 to 53.5)
	NEWS >4	93.3 (84.4 to 100.0)	31.0 (21.1 to 40.8)	32.6 (22.7 to 42.5)	92.9 (83.3 to 100.0)

Legend:

Analysis of Scoring Systems vs outcome measures in patients >85 years old with suspected infection (n=114). PPV = Positive predictive value, NPV = negative predictive value. PPV, NPV, Sensitivity, and Specificity show percentage and 95% confidence intervals.

For peer review only - http://bmjopen.bmj.com/site/about/guidelines.xhtml

Supplementary Figure 1. ROC Curves showing the discriminating ability of the different sepsis scoring systems for escalated care for infection

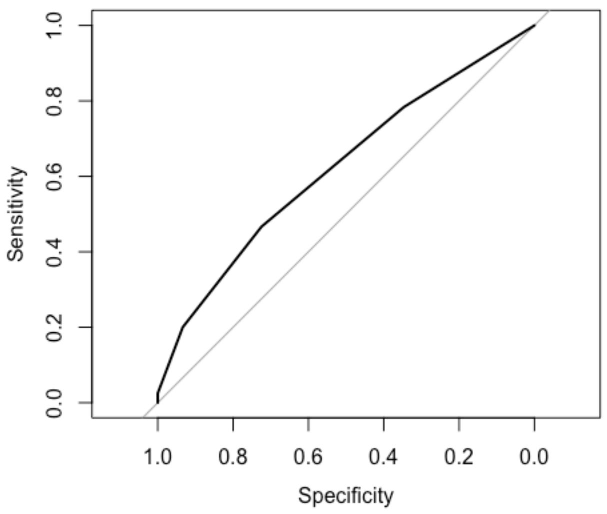

1a SIRS Area Under Curve (AUC) = 0.623 (95% CI: 0.5607-0.6843)

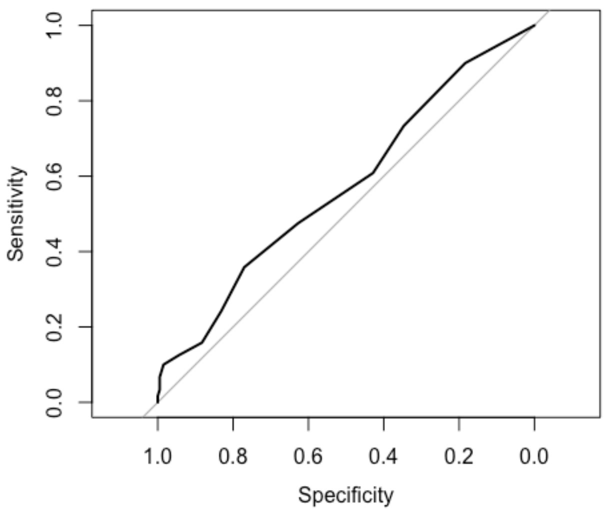

1b NEWS AUC = 0.573 (95% CI: 0.5079-0.6375)

For peer review only - http://bmjopen.bmj.com/site/about/guidelines.xhtml

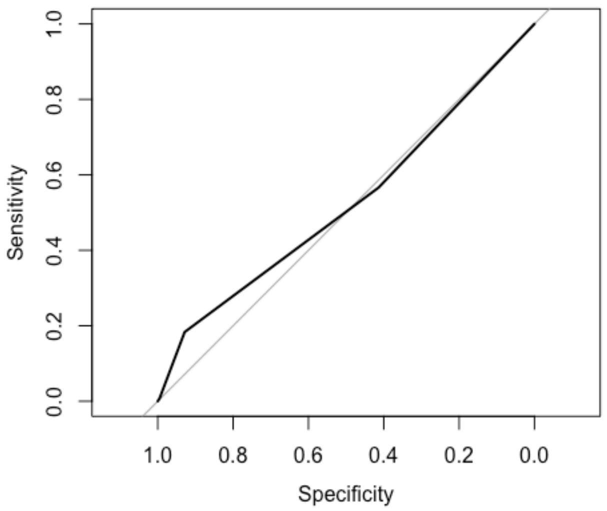

1c qSOFA AUC = 0.523 (95% CI: 0.4612-0.5855)

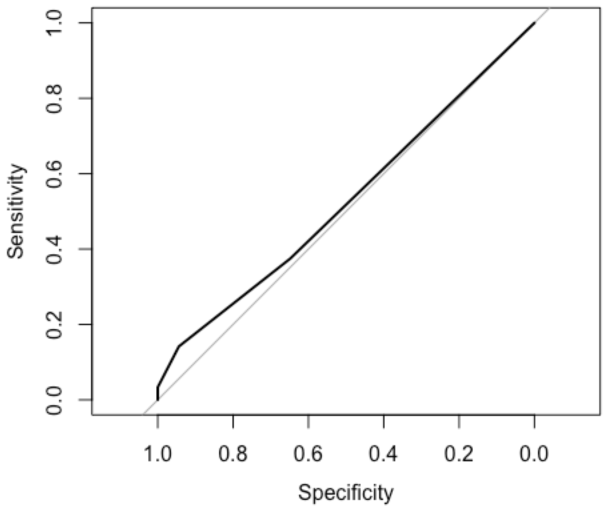

1d NICE High Risk AUC = 0.527 (95% CI: 0.4696-0.584)

For peer review only

For peer review only - http://bmjopen.bmj.com/site/about/guidelines.xhtml

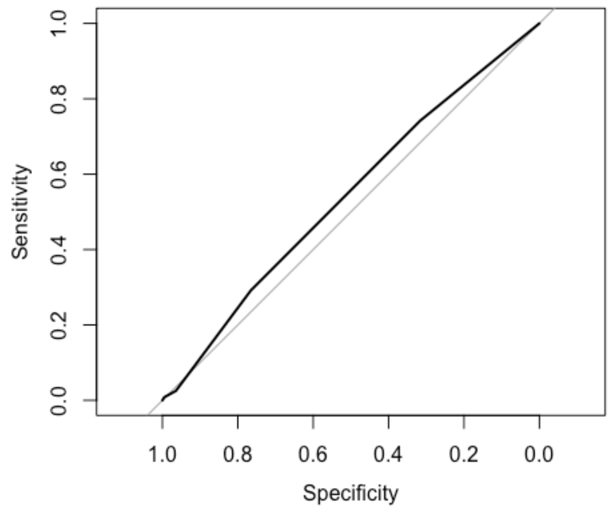

1e NICE Moderate and High Risk AUC = 0.540 (95% CI: 0.4784-0.6006)

For peer review only - http://bmjopen.bmj.com/site/about/guidelines.xhtml

BMJ Open

Sepsis recognition tools in acute ambulatory care: associations with process of care and clinical outcomes in a service evaluation of an Emergency Multidisciplinary Unit in Oxfordshire

Journal:	*BMJ Open*
Manuscript ID	bmjopen-2017-020497.R2
Article Type:	Research
Date Submitted by the Author:	07-Mar-2018

Complete List of Authors:	Camm, Christian; University of Oxford, Radcliffe Department of Medicine<br>Hayward, Gail; University of Oxford, Nuffield Department of Primary Care Health Sciences<br>Elias, Tania; Oxford University Hospitals NHS Foundation Trust, Geratology<br>Bowen, Jordan; Oxford University Hospitals NHS Foundation Trust, Geratology<br>Hassanzadeh, Roya; Imperial College London, Public Health and Primary Care<br>Fanshawe, Thomas; University of Oxford, Nuffield Department of Primary Care Health Sciences<br>Pendlebury, Sarah; Stroke Prevention Reseacrh Unit, Nuffield Department of Clinical Neurosciences; NIHR Oxford Biomedical Research Centre<br>Lasserson, Daniel; University of Birmingham, Institute of Applied Health Research; University of Oxford, Nuffield Department of Medicine

**Primary Subject Heading**:	Health services research
Secondary Subject Heading:	Diagnostics, General practice / Family practice, Infectious diseases
Keywords:	EPIDEMIOLOGY, GENERAL MEDICINE (see Internal Medicine), GERIATRIC MEDICINE, Quality in health care < HEALTH SERVICES ADMINISTRATION & MANAGEMENT

SCHOLARONE™
Manuscripts

Sepsis recognition tools in acute ambulatory care: associations with process of care and clinical outcomes in a service evaluation of an Emergency Multidisciplinary Unit in Oxfordshire

C Fielder Camm[1], Gail Hayward[2], Tania C N Elias[3,4], Jordan S T Bowen[3,4], Roya Hassanzadeh[3,5], Thomas Fanshawe[2], Sarah T Pendlebury,[4,6,7] Daniel S Lasserson[3,4,8,9]

[1]Radcliffe Department of Medicine, University of Oxford, Oxford, UK,

[2]Nuffield Department of Primary Care Health Sciences, University of Oxford, Oxford, UK

[3]Emergency Multidisciplinary Unit, Abingdon Hospital, Oxford Health NHS Foundation Trust, Oxford, UK

[4]Department of Geratology, Oxford University Hospitals NHS Foundation Trust, Oxford, UK

[5]Department of Public Health and Primary Care, Imperial College London, London, UK

[6]Centre for Prevention of Stroke and Dementia, Nuffield Department of Clinical Neurosciences, University of Oxford, Oxford UK.

[7]NIHR Oxford Biomedical Research Centre, John Radcliffe Hospital Oxford, UK.

[8]Nuffield Department of Medicine, University of Oxford, Oxford, UK

[9]Institute of Applied Health Research, College of Medical and Dental Sciences, University of Birmingham, Birmingham, UK

Correspondence to:

Prof Daniel Lasserson, Professor of Ambulatory Care, Murray Learning Centre, Institute of Applied Health Research, College of Medical and Dental Sciences, University of Birmingham, Edgbaston, Birmingham, B15 2TT.

D.S.Lasserson@bham.ac.uk

Manuscript Word Count: 3005 Words

For peer review only

Abstract

Objective

To assess the performance of currently available sepsis recognition tools in patients referred to a community based acute ambulatory care unit.

Design

Service evaluation of consecutive patients over a four month period.

Setting

Community based acute ambulatory care unit

Data collection and outcome measures:

Observations, blood results, and outcome data were analysed from patients with a suspected infection. Clinical features at first assessment were used to populate sepsis recognition tools including: systemic inflammatory responses syndrome (SIRS) criteria, national early warning score (NEWS), quick sequential organ failure assessment (qSOFA), and National Institute for Health and Care Excellence (NICE) criteria. Scores were assessed against the clinical need for escalated care (use of IV antibiotics, fluids, ongoing ambulatory care or hospital treatment) and poor clinical outcome (all-cause mortality and readmission at 30 days after index assessment).

Results

Of 533 patients (median age 81 years), 316 had suspected infection with 120 patients requiring care escalated beyond simple community care. SIRS had the highest positive predictive value (50.9%, 95% confidence interval (CI) [41.6% to 60.3%]) and negative predictive value (68.9%, 95% CI [62.6% to 75.3%]) for the need for escalated care. Both NEWS and SIRS were better at predicting the need for escalated care than qSOFA and NICE criteria in patients with suspected infection (all $p<0.001$). While new onset confusion predicted the need for escalated care for infection in patients ≥85 years old (n= 114), 23.7% of patients ≥85 years had new onset confusion without evidence for infection.

Conclusions

Acute ambulatory care clinicians should use caution in applying the new NICE endorsed criteria for determining need for intravenous therapy and hospital based location of care. NICE criteria have poorer performance when compared against NEWS and SIRS and new onset confusion was prevalent in patients aged ≥ 85 years without infection.

For peer review only

Strengths and limitations of this study

- Acute ambulatory care is applicable to an increasingly prevalent healthcare delivery model
- Consecutive patient evaluation to minimise selection bias
- Not all elements of the sepsis recognition models were available for scoring

Key Words

General Medicine, Geriatric Medicine, Quality in Healthcare, Epidemiology

For peer review only

Introduction

Sepsis, defined as 'life-threatening organ dysfunction caused by dysregulated host response to infection',[1] has a high mortality[2] but early recognition allows for prompt and effective treatment.[3] Therefore, tools for accurate recognition are needed at the earliest possible point after patients attend an initial healthcare assessment setting.

The Third International Consensus (SEPSIS-3) advocates the Sequential Organ Failure Assessment (SOFA)[1] score to evaluate sepsis. However, SOFA is not practical to use in the community and the Quick Sequential Organ Failure Assessment (qSOFA) score has been developed for use in rapid triage and in settings without access to blood tests.[4] The National Institute of Health and Care Excellence (NICE) has recently introduced new guidance regarding sepsis, including a tool for the diagnosis and triage of patients with suspected sepsis in community settings.[2] The use of the qSOFA, and the NICE high-risk and moderate risk criteria (NICE-HR and NICE-MR respectively) are a significant departure from the older systemic inflammatory response syndrome (SIRS) criteria.[5] In addition to sepsis recognition, the National Early Warning Score (NEWS) has been advocated by the Royal College of Physicians to track the clinical condition of patients presenting acutely for hospital and community assessment.[6]

New tools for sepsis recognition have not been evaluated in all healthcare environments that provide initial clinical assessments. Lower sensitivity or specificity of either qSOFA or NICE criteria compared with existing tools for detecting sepsis at initial assessment, particularly in the community, could result in poorer care overall. Lower sensitivity results in missed cases of sepsis compared with current recognition tools and lower specificity results in the unnecessary escalation of care including rapid transfer from community settings to emergency departments, unnecessary use of broad spectrum antibiotics and rapid fluid administration.

The performance of newer recognition scores, such as qSOFA and the NICE criteria, may vary with clinical setting. Low specificity of NICE criteria has been suggested by the large number of acute medical unit admissions it detects as needing rapid assessment within one hour, in comparison with qSOFA and SIRS.[7,8] Furthermore, among emergency department patients with low qSOFA scores, a significant proportion require critical care interventions which would not have been anticipated using qSOFA[9] and the now abandoned SIRS criteria have shown more accurate prediction of death than qSOFA in some emergency department cohorts.[10] Given that in intensive care unit patients, SOFA has a better performance than SIRS,[11,12] it is likely that the clinical setting and nature of the patient cohorts in which these tools are applied have a strong influence on their accuracy to detect sepsis and stratify patients based on risk of poor outcomes.

The acute ambulatory care model, which is recommended as a strategy in acute medicine to meet increasing demands for care within constrained resources,[13] represents a particular challenge for the recognition of sepsis. Whilst ambulatory care is becoming increasingly prevalent,[14] there are very few tools designed to support clinical decision making in the ambulatory care environment. Patients who are deemed suitable for ambulatory care do not have very disordered

For peer review only - http://bmjopen.bmj.com/site/about/guidelines.xhtml

physiology[15], even though there may be an emerging underlying significant disease process. Furthermore, identification of sepsis in older patients with frailty is complex as acute functional decline has many other causes,[16] yet mortality and morbidity related to sepsis is higher in the older population.[17] One of the critical decisions in ambulatory care management of suspected infection is selecting which patients can be safely discharged with oral therapy and follow up from their general practitioner and which patients are at higher risk of clinical deterioration and require care that is enhanced, either through an ambulatory platform with daily parenteral antibiotics with senior clinical review, or medical admission.

The ambulatory care clinician, faced with the diagnostic and management challenge of early sepsis recognition and the need to safely manage patients on an ambulatory pathway, can choose from four different sepsis recognition tools (SIRS, NEWS, NICE-HR/MR, and qSOFA). This study aims to evaluate the performance of these tools by examining their association with processes of care and clinical outcomes in patients with suspected infection in an acute ambulatory care setting. Given the uncertainty about application to older people in whom there is a wide differential diagnosis for acute frailty syndromes such as confusion, we undertook a subgroup analysis in patients 85 years or older.

Methods

The direct care team collected healthcare data as part of routinely provided healthcare from consecutive patients presenting to Abingdon Hospital's Emergency Multidisciplinary Unit (EMU) run by Oxford Health NHS Foundation Trust between 12/08/2015 and 08/12/2015. Patients were included if they were resident within Oxfordshire, allowing for appropriate follow-up details to be obtained, and had a clinical phenotype suggesting possible infection, in keeping with the recommended application of sepsis screening tools.[5] The use of routine healthcare data collected by the direct care team for the purposes of determining adherence to national guidance and application of clinical prediction tools to inform future service development was prospectively approved as a service evaluation by Oxford University Hospitals NHS Foundation Trust (providers of the medical care on EMU), with audit datix reference number 3812, and permission to publish was granted by Oxford Health NHS Foundation Trust R&D Department.

Study setting:

The EMU model of care provides assessment and treatment for adults with acute illness where an ambulatory treatment path is considered likely by the initial contact healthcare provider. Facilities include point of care blood testing (electrolytes, renal function, blood gases, lactate, C-reactive protein, troponin, INR), plain X Ray, and intravenous delivery of medications and fluids. Referrals are primarily from general practitioners and paramedic teams in the community. Patients whose care needs cannot be met with an ambulatory pathway are admitted to either a community hospital or an associated acute hospital.

For peer review only

Process of care and clinical outcomes:

Details regarding the patients' presentation, investigations, treatment, and outcome were prospectively documented at the time of assessment on the EMU. Patients' initial assessment was undertaken using a structured clinical clerking proforma including a brief cognitive test (the abbreviated mental test score (AMTS) and delirium screen) as described previously.[18] Data for analyses were extracted from the clerking proforma supplemented by information from ambulance sheets, GP referral letters, and communications with patients and relatives as listed in Supplementary Table 1. Patients were considered to have a possible infection if a clinical suspicion of infection was recorded by the treating clinician or if the presenting complaint suggested an infective cause for their symptoms. Processes of care included use of intravenous (IV) antibiotics, IV fluids and pathway of care (ambulatory vs hospital) and re-admission within 30 days. Clinical outcome was 30-day all-cause mortality.

We tested the ability of sepsis scoring systems to select patients who require continued monitoring and treatment from the medical team, either via ambulatory or bed based pathways. Patients were considered to have required escalated care for an infection if *all* the following conditions were met: (1) the patient had a possible infection, (2) the patient received IV antibiotics and IV fluids, (3) the patient was either admitted to an inpatient facility or subsequently brought back to the EMU for ≥1 daily reviews, or enrolled in the 'hospital @ home' programme to receive ongoing IV antibiotics and daily review after initial EMU assessment. Furthermore, patients with suspected infection who did not meet conditions (2) and (3) were considered to have needed escalated care if they died within 7-days of review or required re-admission for infection within 30 days. This strategy was used as it collectively identifies patients for whom initial discharge from ambulatory care with no or oral antibiotics may have been inappropriate.

Sepsis Scoring Systems:

We evaluated the accuracy of SIRS,[18] qSOFA,[6] NEWS,[2] NICE-HR and NICE-MR from NICE Guideline NG51[7] for detecting the need for escalated care to treat suspected bacterial infection. Each method was undertaken in accordance with guidance provided by issuing bodies. Features associated with these tools available in our data-set and cut-off values for prediction of sepsis or poor outcome are detailed in Supplementary Table 2. Within selected scoring systems, (NICE-MR, NICE-HR), some variables are not systematically available at the first assessment in an acute setting (e.g. urine output, usual blood pressure values for calculation of difference from observed value) and Supplementary Table 2 lists these variables together with any additional clinical features that were not systematically sought within the clerking proforma. The scores were calculated without use of these items which would reflect real world practice after implementation. A cut-off score of >4 was used for NEWS in line with guidance from the Royal College of Physicians (London) which details this threshold for separating low-risk patients from those at increased risk.[7] For cases in which individual items required for a score were

For peer review only

missing, the score for that individual was calculated without that item, in line with clinical practice. During the analysis NICE-MR and HR were combined in some analyses to replicate the use of the moderate criteria in clinical practice. We did not separately analyse the Sepsis Trust Red/Amber flag system (https://sepsistrust.org/education/clinical-tools/) as features of this system were incorporated into the new NICE guidelines.

Statistical Analysis:

Continuous variables are expressed as either mean (standard deviation) or median (interquartile range [IQR]) and compared across suspected infection/no suspected infection and escalated care/no escalated care groups using the independent student's T-test or Mann-Whitney U-test respectively. Categorical and ordinal variables are reported as frequency (percentage) and compared between groups using the χ^2 or Fisher's exact test. The diagnostic accuracy of each predictive tool was assessed using positive predictive value (PPV), negative predictive value (NPV), sensitivity and specificity, with 95% confidence intervals. Predictive tools were compared with one another using McNemar's test, and we consider p-values of less than 0.005 to be statistically significant in these comparisons (based on a conservative Bonferroni correction for ten comparisons). The performance of sepsis tools was assessed in the whole dataset and in a subgroup of the oldest patients (≥85 years) in predicting the need for escalated care, mortality, and readmission within 30 days of index assessment. Analysis was performed using Version 9 of the SAS system for Windows. Copyright © 2002-2010 by SAS Institute Inc., Cary, NC, USA.

Patient involvement:

This study was a service evaluation. There was no direct patient involvement in its conduct.

Results

A total of 533 patients were assessed during the observation time period. Ten patients were not local residents and did not have follow-up data and were excluded from the analysis (Figure 1). Demographic data of the study population are provided in Table 1. 316 patients were considered to have a potential infection with 120 patients requiring escalated treatment for infection. Features most significantly associated with need for escalated care were increased heart rate (89.6 (18.9) vs. 83.8 (17.8), p=0.006), increased temperature (37.0 (0.9) vs 36.6 (0.6), p<0.001), and increased white cell count (14.5 (17.4) vs. 9.3 (3.7), p=0.003). In the whole cohort, there was no association between new onset confusion and need for escalated care (22/120 (20.0%) vs. 27/196 (14.0%), p=0.172). A full list of features associated with escalated care is displayed in Table 1.

Of 186 patients ≥85 years old (45% of the cohort), 114 patients had a suspected infection as the cause for their presentation. Among patients ≥85 years with suspected infection, those who met criteria for escalated care (n=35, 30.7%)

For peer review only

also demonstrated the positive findings for the whole cohort in terms of difference in physiological markers, but new confusion was significantly associated with escalated care in this subgroup (Table 1).

The PPV, NPV, sensitivity and specificity of each assessment tool against the need for escalated care, 30-day mortality, 30-day admission, and a composite marker of 30-day mortality or admission for the cohort with suspected infection are shown in Table 2. SIRS had the highest PPV (50.9%, 95% CI [41.6% to 60.3%]) and NPV (68.9%, 95% CI [62.6% to 75.3%]). Using McNemar's test, the combined high- and moderate-risk NICE criteria were shown to be significantly worse at predicting escalated care compared with SIRS ($p<0.001$), NEWS ($p<0.001$), and NICE High-risk criteria alone ($p=0.004$) (Table 3), a result driven by very low specificity of the combined high- and moderate-risk NICE criteria (21.4%, 95% CI [15.7 to 27.2]). Furthermore, both SIRS and NEWS were shown to have better performance against qSOFA (both $p<0.001$). NEWS and SIRS had the highest specificity for detecting 30-day mortality (67.0 95% CI [61.6 to 72.4] and 73.5 95% CI [68.5 to 78.6] respectively).

Among patients ≥85 years old, the PPV and NPV of both SIRS and NEWS were higher than NICE or qSOFA scores for escalated care and for the combined 30 day clinical outcome (Supplementary Table 3). Whilst NICE criteria had the highest sensitivity at detecting poor clinical outcomes (mortality or admission within 30 days) (88.4%), this was at the cost of very low specificity (14.3%). Receiver operator characteristic curves for the ability of the different sepsis scoring systems to identify patients requiring escalated care are shown in Supplementary Figure 1.

Discussion

Of the tools assessed, SIRS and NEWS were shown to have the highest PPV and NPV for escalated care in the dataset overall and in the subgroup of patients ≥ 85 years. Whilst NICE criteria and qSOFA had better performance in detecting requirement for escalated care in patients ≥85 years, their poorer specificity contributed to a lower PPV. The presence of new confusion was associated with escalated care among patients with suspected infection in the subgroup of patients aged ≥ 85 years.

Although SIRS was shown to be the most predictive of escalated care in patients with suspected infection in this study, the combined high and moderate risk NICE criteria would capture the most patients requiring escalated care but at the cost of very low specificity i.e. a high false positive rate. These results contrast with the previously suggested limitations in predictive ability for poor outcomes when compared against other potential sepsis recognition and stratification tools which have led to a move away from using SIRS.[5] This may reflect applicability in different patient cohorts as Kaukonen *et al.* demonstrated that 12.1% of intensive care unit patients with severe sepsis did not meet ≥2 SIRS criteria.[2 19 20] However, they also showed that those with SIRS positive sepsis had significantly increased mortality compared with those not

For peer review only

meeting these criteria. Churpek *et al.* demonstrated that 47% of an acute patient cohort met ≥2 SIRS criteria during admission and concluded that screening patients for sepsis based on these criteria would be unwise.[19] However, this paper did not select patients in whom infection was suspected and instead applied it to all admissions, irrespective of presenting complaint. A recent analysis from the same group has also suggested that in those admitted to hospital and suspected of infection, discrimination for in-hospital mortality was highest using NEWS.[21] Our results add to this information by providing support for the use of NEWS in identifying patients at need of care escalation in a community setting.

The importance of identifying sepsis in the initial stages of clinical contact to allow for prompt and effective treatment has been well documented.[20] However, our results have shown that those systems endorsed by both NICE and Sepsis-3 have limitations related to high false-positive rates as compared with both SIRS and NEWS, which could lead to substantial over-treatment.

The introduction of new-onset confusion as a predictor of sepsis in NICE guidance risks increasing the use of inappropriate antibiotics in the older patient with frailty. Although our results show a significant association between the development of new confusion and the need for escalated care among patients ≥ 85 years with infection, there was a high prevalence of new onset confusion in this age group without any evidence of underlying infection. While infection is a principle cause of delirium,[3] there is limited high-quality evidence that altered mental state in older adults is predictive of sepsis itself [22] and delirium has a wide range of potential causes in older people.[5]

Our data show that the new combined NICE high-risk and moderate-risk criteria are likely to significantly increase the number of patients that are determined as potentially having sepsis at initial assessment in ambulatory care. As such, this is likely to increase the number of patients receiving intravenous antibiotics which may conflict with the national and global priorities of antibiotic stewardship.[22] Furthermore, in the ambulatory care setting their introduction may increase conversion to formal hospital admission which our data suggest will not be justified based on the clinical outcomes we recorded.

Limitations

Several limitations of this work are acknowledged. Data collection for this work was undertaken at a time when SIRS was the recommended assessment tool for potential sepsis. As such, incorporation bias may explain some degree of the correlation between this method of assessment and the diagnosis and treatment of sepsis. However, SIRS was not calculated as part of the standard assessment proforma in this cohort and white cell count was not available at initial review as it is not part of the point of care testing portfolio. This limitation would not explain the correlation between SIRS and NEWS with mortality at 30-days.

Not all elements of the new NICE criteria for high- and moderate-risk sepsis were available for this analysis. However, the limited specificity of both these methods in determining sepsis would only be worsened by the addition of further criteria

For peer review only

to this analysis. Furthermore, we argue that several of those unavailable elements (e.g. Mottled Skin, Cyanosis, and Urine Output) represent those that would either not be available in the acute ambulatory setting or are based on subjective assessment and therefore more likely to vary between clinicians.

Conclusions:

This study demonstrates that new NICE recommended sepsis tools do not outperform the existing tools in an ambulatory care setting in predicting the need for escalated care for infection and clinical outcomes at 30 days. Although NICE tools had higher sensitivity, this was at the expense of very low specificity. SIRS had the highest predictive values but the requirement of a white cell count implies that it can only be used widely if this is tested at the point of care. Further research should be undertaken to determine applicability of NICE sepsis guidance in different healthcare settings where acutely unwell patients are assessed before widespread adoption in the NHS.

For peer review only

Funding Statement

CFC is funded by an NIHR Academic Clinical Fellowship. The NIHR Oxford Biomedical Research Centre supported DSL and SP with grant number AC14/035 supporting JSTB and TCNE. JSTB is also supported by the NIHR Oxford Collaboration for Leadership in Applied Health Research and Care (CLAHRC) at Oxford Health NHS Foundation Trust. This article presents independent research funded by the NIHR Community Healthcare MedTech and Invitro Diagnostic Co-operative. The opinions expressed in this study are those of the authors and not necessarily those of the NIHR, the Department of Health or the NHS.

Contribution Statement

CFC undertook analyses and drafted the initial manuscript. GH and TF advised on analytical methodology, interpreted data and critically revised the manuscript. JSTB, TCNE and RH designed data collection methods, collected data, clinically interpreted analyses and critically revised the manuscript. SP designed the service evaluation, data collection methods and critically revised the manuscript. DSL designed the service evaluation, advised on analytical methods, interpreted data and critically revised the manuscript.

Ethical approval

This study was part of a service evaluation of the care delivered by the Emergency Multidisciplinary Unit, with routinely collected data used to determine future care design and use of appropriate clinical decision support. The service evaluation was prospectively approved by the Oxford University Hospitals NHS Foundation Trust with Datix reference number 3812. The evaluation was also approved by Oxford Health NHS Foundation Trust Older People's Directorate Audit Department and permission to publish was granted by Oxford Health NHS Foundation Trust Research and Development Department. As this study had approval as a service evaluation, we did not seek formal ethical approval through the national research ethics service.

Data sharing statement

No additional data are available

Conflict of Interest

None declared.

For peer review only

References

1. Fleischmann C, Scherag A, Adhikari NK, et al. Assessment of Global Incidence and Mortality of Hospital-treated Sepsis. Current Estimates and Limitations. Am J Respir Crit Care Med 2016;**193**(3):259-72.

2. Singer M, Deutschman CS, Seymour CW, et al. The Third International Consensus Definitions for Sepsis and Septic Shock (Sepsis-3). JAMA 2016;**315**(8):801-10.

3. Ferrer R, Martin-Loeches I, Phillips G, et al. Empiric antibiotic treatment reduces mortality in severe sepsis and septic shock from the first hour: results from a guideline-based performance improvement program. Crit Care Med 2014;**42**(8):1749-55.

4. Vincent JL, Moreno R, Takala J, et al. The SOFA (Sepsis-related Organ Failure Assessment) score to describe organ dysfunction/failure. On behalf of the Working Group on Sepsis-Related Problems of the European Society of Intensive Care Medicine. Intensive Care Med 1996;**22**(7):707-10.

5. National Institute of Health and Care Excellence. NICE Guideline 51 - Sepsis: recognition, assessment and early management: National Institute of Health and Care Excellence, 2016.

6. Bone RC, Balk RA, Cerra FB, et al. Definitions for Sepsis and Organ Failure and Guidelines for the Use of Innovative Therapies in Sepsis. Chest 1992;**101**(6):1644-55.

7. National Early Warning Score (NEWS). Standardising the assessment of acute-illness severity in the NHS: Royal College of Physicians (London), 2012.

8. Smith GB, Prytherch DR, Schmidt PE, et al. Review and performance evaluation of aggregate weighted 'track and trigger' systems. Resuscitation 2008;**77**(2):170-9.

9. Thompson A, Stevens M, Collin I, et al. Evolving sepsis definitions and their impact on Acute Medical Units. Acute medicine 2017;**16**(1):25-29.

10. Moskowitz A, Patel PV, Grossestreuer AV, et al. Quick Sequential Organ Failure Assessment and Systemic Inflammatory Response Syndrome Criteria as Predictors of Critical Care Intervention Among Patients With Suspected Infection. Crit Care Med 2017.

11. Askim A, Moser F, Gustad LT, et al. Poor performance of quick-SOFA (qSOFA) score in predicting severe sepsis and mortality - a prospective study of patients admitted with infection to the emergency department. Scandinavian journal of trauma, resuscitation and emergency medicine 2017;**25**(1):56.

12. Williams JM, Greenslade JH, McKenzie JV, et al. Systemic Inflammatory Response Syndrome, Quick Sequential Organ Function Assessment, and Organ Dysfunction: Insights From a Prospective Database of ED Patients With Infection. Chest 2017;**151**(3):586-96.

13. Commission FH. Future hospital: caring for medical patients. A report from the Future Hospital Commission to the Royal College of Physicians. London, 2013.

14. Nasa P, Juneja D, Singh O, et al. Severe sepsis and its impact on outcome in elderly and very elderly patients admitted in intensive care unit. J Intensive Care Med 2012;**27**(3):179-83.

15. Raith EP, Udy AA, Bailey M, et al. Prognostic Accuracy of the SOFA Score, SIRS Criteria, and qSOFA Score for In-Hospital Mortality Among Adults With Suspected Infection Admitted to the Intensive Care Unit. Jama 2017;**317**(3):290-300.

16. Ala L, Mack J, Shaw R, et al. Selecting ambulatory emergency care (AEC) patients from the medical emergency in-take: the derivation and validation of the Amb score. Clinical medicine (London, England) 2012;**12**(5):420-6.

17. Girard TD, Opal SM, Ely EW. Insights into severe sepsis in older patients: from epidemiology to evidence-based management. Clin Infect Dis 2005;**40**(5):719-27.

18. Pendlebury ST, Lovett NG, Smith SC, et al. Observational, longitudinal study of delirium in consecutive unselected acute medical admissions: age-specific rates and associated factors, mortality and re-admission. BMJ Open 2015;**5**(11):e007808.

For peer review only

19. Kaukonen KM, Bailey M, Pilcher D, et al. Systemic inflammatory response syndrome criteria in defining severe sepsis. N Engl J Med 2015;**372**(17):1629-38.

20. Churpek MM, Zadravecz FJ, Winslow C, et al. Incidence and Prognostic Value of the Systemic Inflammatory Response Syndrome and Organ Dysfunctions in Ward Patients. Am J Respir Crit Care Med 2015;**192**(8):958-64.

21. Churpek MM, Snyder A, Han X, et al. Quick Sepsis-related Organ Failure Assessment, Systemic Inflammatory Response Syndrome, and Early Warning Scores for Detecting Clinical Deterioration in Infected Patients outside the Intensive Care Unit. Am J Respir Crit Care Med 2017;**195**(7):906-11.

22. Fong TG, Tulebaev SR, Inouye SK. Delirium in elderly adults: diagnosis, prevention and treatment. Nat Rev Neurol 2009;**5**(4):210-20.

For peer review only

Figure 1 Patient flow from clinical presentation to outcomes

Tables

Table 1:

Item	All patients with suspected infection				≥85 years old				<85 years old			
		Need for escalated care				Need for escalated care				Need for escalated care		
	N	Yes	No	p-value	N	Yes	No	p-value	N	Yes	No	p-value
Subjects	316	120 (38.0%)	196 (62.0%)	.	114	35 (30.7%)	79 (69.3%)	.	202	85 (42.1%)	117 (57.9%)	.
Proportion Male	316	57 (47.5%)	79 (40.3%)	0.210	114	10 (28.6%)	33 (41.8%)	0.180	202	47 (55.3%)	46 (39.3%)	0.024
Age (years)	316	74.5 (16.6)	75.2 (18.3)	0.708	114	90.2 (3.3)	89.7 (3.9)	0.442	202	68.0 (15.5)	65.4 (17.7)	0.283
Heart Rate (beats min^{-1})	315	89.6 (18.9)	83.8 (17.8)	0.006	114	89.5 (19.2)	79.29 (15.5)	0.003	201	89.6 (18.9)	86.9 (18.6)	0.306
Respiratory Rate	314	21.6 (6.4)	21.3 (5.3)	0.643	113	24.4 (8.2)	20.7 (5.2)	0.016	201	20.5 (5.1)	21.7 (5.4)	0.094
Systolic Blood Pressure	314	132.2 (20.1)	135.9 (26.7)	0.172	113	129.1 (21.4)	132.0 (26.1)	0.534	201	133.5 (19.5)	138.5 (26.9)	0.133
Oxygen Saturations (%)	313	96 (94 - 98)	97 (95 - 98)	0.007	113	96 (93 - 97)	97 (95 - 98)	0.009	200	96 (94 - 98)	97 (94 - 98)	0.132
Temperature (°C)	314	37.0 (0.9)	36.6 (0.6)	<0.001	113	36.9 (0.9)	36.5 (0.6)	0.044	201	37.1 (0.9)	36.7 (0.6)	0.001
AMTS	303	10 (8 - 10)	10 (9 - 10)	0.155	107	7 (5 - 9)	9 (7 - 10)	0.008	196	10 (9 - 10)	10 (9 - 10)	0.532
Known Dementia Diagnosis	316	21 (17.5%)	23 (11.7%)	0.151	114	9 (25.7%)	14 (17.7%)	0.327	202	12 (14.1%)	9 (7.7%)	0.140
New Confusion	316	22 (18.3%)	27 (13.8%)	0.172	114	15 (42.9%)	18 (22.8%)	0.012	202	7 (8.2%)	9 (7.7%)	0.769
White Cell Count(x 10^9/L)	274	14.5 (17.4)	9.3 (3.7)	0.003	99	13.9 (6.4)	9.3 (4.1)	0.001	175	14.7 (20.4)	9.3 (3.3)	0.028
Mortality (30-Day)	316	14 (11.7%)	11 (5.6%)	0.053	114	8 (22.9%)	5 (6.3%)	0.010	202	6 (7.1%)	6 (5.1%)	0.567
Barthel Score ≥15	316	80 (66.7%)	150 (76.5%)	0.056	114	17 (48.6%)	53 (67.1%)	0.061	202	63 (74.1%)	97 (82.9%)	0.129

Sepsis scores at initial presentation									
NEWS	316	3 (1 – 5)	3 (1 – 4)	114	4 (3 – 9)	3 (1 – 4)	202	3 (1 – 5)	3 (1 – 5)
SIRS criteria	316	1 (1 – 2)	1 (0 – 2)	114	2 (1 – 2)	1 (0 – 1)	202	1 (0 – 2)	1 (0 – 2)
qSOFA criteria	316	1 (0 – 1)	1 (0 – 1)	114	1 (1 – 2)	1 (0 – 1)	202	0 (0 – 1)	1 (0 – 1)
NICE High-Risk	316	45 (37.5%)	69 (35.2%)	114	21 (60.0%)	31 (39.2%)	202	24 (28.2%)	38 (32.5%)
Nice Moderate- and High-Risk	316	96 (80.0%)	154 (78.6%)	114	31 (88.6%)	69 (87.3%)	202	65 (76.5%)	85 (72.7%)

Legend:

Demographic characteristics of patients assessed on the EMU during this analysis with suspected infection (n=316). AMTS – Abbreviated Mental Test Score. Results displayed as N(%), mean (standard deviation), or median(inter-quartile range) unless otherwise indicated. For the scoring systems evaluated, all data points evaluated were available in the following numbers: SIRS (n=272), NICE High-Risk (n=314), NICE Moderate- and High-Risk (n=314), qSOFA (n=314), NEWS (n=313).

For peer review only

Table 2

Outcome Measure	*Test*	*PPV (%)*	*NPV (%)*	*Sensitivity (%)*	*Specificity (%)*
**Escalated care**					
	SIRS	50.9 (41.6 to 60.3)	68.9 (62.6 to 75.3)	46.7 (37.7 to 55.6)	72.4 (66.2 to 78.7)
	NICE High-Risk	39.5 (30.5 to 48.4)	62.9 (56.2 to 69.5)	37.5 (28.8 to 46.2)	64.8 (58.1 to 71.5)
	Nice Moderate- and High-Risk	38.4 (32.4 to 44.4)	63.6 (52.0 to 75.2)	80.0 (72.8 to 87.2)	21.4 (15.7 to 27.2)
	qSOFA	37.2 (30.2 to 44.2)	60.9 (52.6 to 69.2)	56.7 (47.8 to 65.5)	41.3 (34.4 to 48.2)
	NEWS >4	48.9 (38.4 to 59.3)	66.2 (60.1 to 72.4)	35.8 (27.3 to 44.4)	77.0 (71.2 to 82.9)
**30-Day Mortality**					
	SIRS	12.7 (6.5 to 19.0)	94.7 (91.6 to 97.7)	56.0 (36.5 to 75.5)	67.0 (61.6 to 72.4)
	NICE High-Risk	12.3 (6.3 to 18.3)	94.6 (91.4 to 97.7)	56.0 (36.5 to 75.5)	65.6 (60.2 to 71.1)
	Nice Moderate- and High-Risk	9.6 (5.9 to 13.3)	98.5 (95.5 to 100.0)	96.0 (88.3 to 100.0)	22.3 (17.6 to 27.1)
	qSOFA	11.5 (6.9 to 16.1)	97.0 (94.1 to 99.9)	84.0 (69.6 to 98.4)	44.3 (38.6 to 50.0)
	NEWS >4	12.5 (5.6 to 19.4)	93.9 (90.7 to 97.0)	44.0 (24.5 to 63.5)	73.5 (68.5 to 78.6)
**30-Day readmission**					
	SIRS	82.7 (75.7 to 89.8)	34.0 (27.5 to 40.4)	40.1 (33.7 to 46.5)	78.7 (70.1 to 87.2)
	NICE High-Risk	74.6 (66.6 to 82.6)	29.7 (23.4 to 36.0)	37.4 (31.1 to 43.7)	67.4 (57.7 to 77.2)
	Nice Moderate- and High-Risk	74.8 (69.4 to 80.2)	39.4 (27.6 to 51.2)	82.4 (77.4 to 87.3)	29.2 (19.8 to 38.7)
	qSOFA	74.9 (68.6 to 81.1)	32.3 (24.4 to 40.3)	60.4 (54.0 to 66.7)	48.3 (37.9 to 58.7)
	NEWS >4	78.4 (69.8 to 87.0)	30.7 (24.7 to 36.7)	30.4 (24.4 to 36.4)	78.7 (70.1 to 87.2)
**30-Day Composite**					
	SIRS	83.6 (76.7 to 90.5)	33.5 (27.1 to 39.9)	40.2 (33.8 to 46.5)	79.3 (70.8 to 87.8)
	NICE High-Risk	75.4 (67.5 to 83.3)	29.2 (22.9 to 35.5)	37.6 (31.3 to 43.8)	67.8 (58.0 to 77.6)
	Nice Moderate- and High-Risk	75.6 (70.3 to 80.9)	39.4 (27.6 to 51.2)	82.5 (77.6 to 87.5)	29.9 (20.3 to 39.5)
	qSOFA	75.4 (69.2 to 81.6)	31.6 (23.7 to 39.5)	60.3 (53.9 to 66.6)	48.3 (37.8 to 58.8)
	NEWS >4	79.5 (71.1 to 88.0)	30.3 (24.3 to 36.2)	30.6 (24.6 to 36.5)	79.3 (70.8 to 87.8)

Legend:

Analysis of Scoring Systems vs outcome measures in patients presenting to the EMU with suspected infection (n=316). PPV = Positive predictive value, NPV = negative predictive value. PPV, NPV, Sensitivity, and Specificity show percentage and 95% confidence intervals.

For peer review only

Table 3:

	NEWS >4	*qSOFA*	*NICE MR and HR*	*NICE High-Risk*
SIRS	0.2 (P=0.893)	21.3 (P<0.001)	25.7 (P<0.001)	6.63 (P=0.01)
NICE High-Risk	5.9* (P=0.034)	5.9 (P=0.015)	8.5 (P=0.004)	.
NICE MR and HR	18.5* (P<0.001)	1.27 (P=0.259)	.	.
qSOFA	19.3* (P<0.001)	.	.	.

Legend:

McNemar's analysis of each predictive tool compared against one another in determining the need for escalated care in patients with suspected infection (n=316).

Nice MR and HR – Combined analysis of NICE moderate- and high-risk criteria.

Values represent row-listed diagnostic tool performed better than column-listed diagnostic tool.

* represents column-listed diagnostic tool performed better than row-listed diagnostic tool.

A p-value of <0.005 is considered significant following Bonferroni correction for multiple analysis.

For peer review only

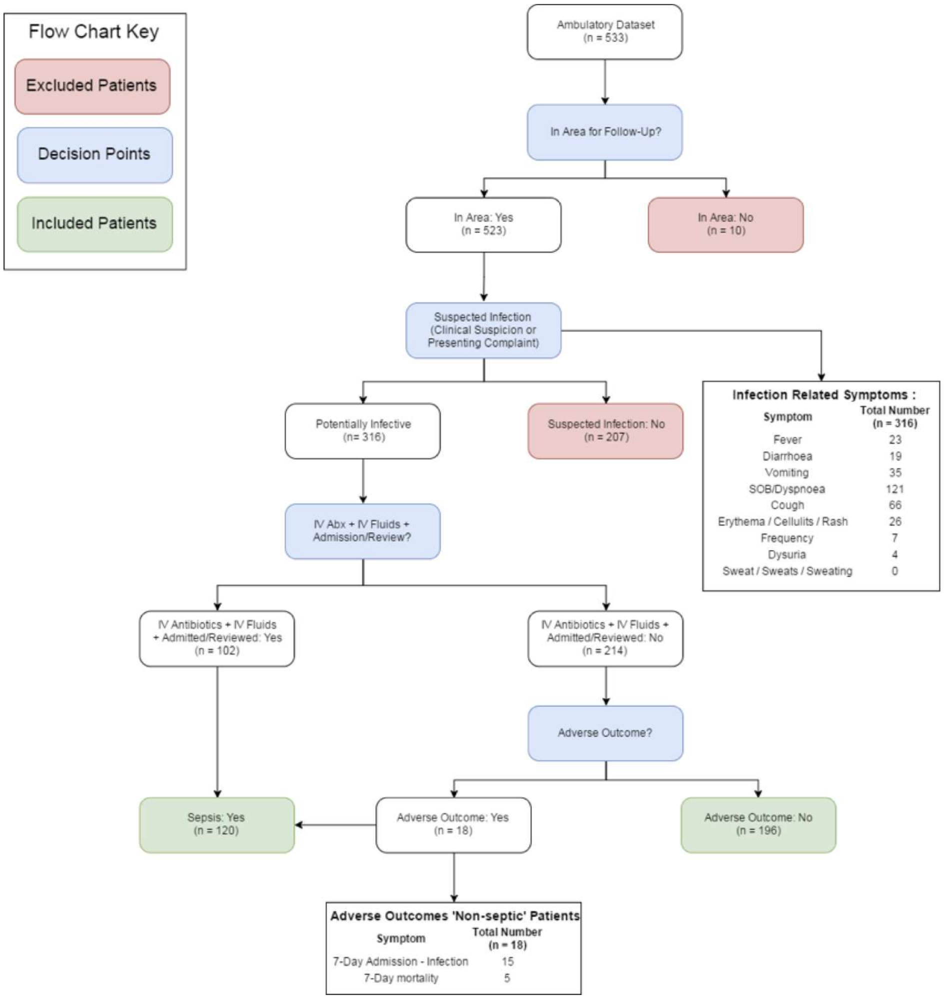

Patient flow from clinical presentation to outcomes

318x314mm (300 x 300 DPI)

Supplementary Material

Supplementary Table 1:

Data Field	*Data type*
**Demographics**	
Study ID	Numerical
Age	Numerical
Sex	Categorical
Weight	Numerical
Height	Numerical
**Initial Attendance Information**	
Date of first attendance	Date
Attendance time	Time
Was the patient accompanied	Boolean
Most senior clinician reviewing patient on initial attendance	Categorical
**Patient Background**	
Comorbidities	Free Text
Known Dementia	Boolean
**Presenting complaints**	
Presenting Complaint	Free Text
Confusion	Boolean
Delirium	Boolean
SOB	Boolean
Confusion / altered behaviour	Boolean
Decreased mobility	Boolean
Increased care needs	Boolean
Falls	Boolean
Chest Pain, tachycardia, or palpitations	Boolean
Other respiratory (cough, wheeze, sore throat)	Boolean
Abdominal symptoms (pain, bloating, diarrhoea, nausea, vomiting, constipation	Boolean
Urinary or GU symptoms (dysuria, catheter problems, retention)	Boolean
Fatigue, weight loss, reduced oral intake	Boolean
Bilateral leg swelling	Boolean
Pain (back, hip/groin, shoulder, knee, wrist, toe, facial)	Boolean
Biochemical or haematological derangement (electrolytes, renal function, LFTs)	Boolean
Symptomatic anaemia	Boolean
Glycaemic derangement	Boolean
Other neurological (seizure, headache, tremor, visual symptoms, virtigo, tinnitus)	Boolean
PR bleed / malaena	Boolean
Leg weakness	Boolean
Fever	Boolean
Rash / erythema (incl face)	Boolean
Unilateral swelling	Boolean
Collapse	Boolean
Drowsiness	Boolean
Dizziness / unsteadiness	Boolean
Anxiety / low mood	Boolean
**Observations on Admission**	
Heart rate	Numerical
Systolic blood pressure	Numerical
Diastolic blood pressure	Numerical
Oxygen saturations	Numerical

For peer review only - http://bmjopen.bmj.com/site/about/guidelines.xhtml

Respiratory rate	Numerical
Temperature	Numerical
Postural drop in blood pressure	Boolean
AMTS	Ordinal
Glasgow coma score	Ordinal
**EMU Blood Results (categories)**	
Full blood count panel	Numerical
Urea and electrolytes panel	Numerical
C-reactive protein	Numerical
Liver function panel	Numerical
INR	Numerical
Lactate	Numerical
Troponin	Numerical
**Initial Diagnosis**	
Initial Diagnosis	Free text
Suspected infection	Boolean
Infection Source	Free text
**Treatments initiated**	
IV fluids	Boolean
Blood transfusion	Boolean
Nebulisers	Boolean
IV antibiotics	Boolean
Oral antibiotics	Boolean
IV iron	Boolean
IV furosemide	Boolean
Medications held	Boolean
Laxatives	Boolean
Analgesia	Boolean
Management	Free Text
**Outcomes**	
Initial plan (ambulation or admission)	Categorical
Escalation unit	Categorical
Documented reason for escalation	Free text
Date of death	Date
Discharge Date	Date
Number of visits to EMU	Numerical
Length of stay (if admitted)	Numerical
Date of EMU review	Date
Reason for EMU review	Free text
Unexpected re-admission within 30 days	Boolean

Legend:

Relevant variables collected as part of routine healthcare provision

For peer review only - http://bmjopen.bmj.com/site/about/guidelines.xhtml

For peer review only

Supplementary Table 2

	Score components		
Method	*Available*	*Unavailable*	*Cut-off value*
**NICE-HR** [1]			
	HR > 130 bpm	Mottled Skin	≥1
	RR > 25 min^{-1}	Cyanosis	
	New oxygen requirement	Non-blanching Rash	
	Systolic BP <90 mmHg	Urine Output	
	New Confusion	Systolic BP 40mmHG below normal	
**NICE-MR** [1]			
	HR 90 – 130 bpm	Steroid use / impaired immunity	≥1
	RR 21-24 min^{-1}	Trauma or Invasive Procedure	
	Systolic BP 90-100 mmHg	Redness/swelling/discharge at surgical site or breakdown of wound	
	Temperature <36°C		
	Reduced mobility or deterioration		
**qSOFA** [2]			
	GCS < 15	-	≥1
	RR > 21 min-1		
	Systolic BP ≤100 mmHg		
**SIRS** [3]			
	WCC <4.0 x10^3 or >12 x 10^3	-	≥2
	HR > 90 bpm		
	Temperature <36°C or >38°C		
	RR > 20 min^{-1}		
**NEWS** [4]			
	HR (multiple thresholds)	-	>4
	RR (multiple thresholds)		
	Oxygen Saturation (multiple thresholds)		
	Supplemental Oxygen (multiple thresholds)		
	Temperature (multiple thresholds)		
	Systolic BP (multiple thresholds)		
	Level of Consciousness (AVPU Scale)		

Legend

Components of diagnostic and severity assessment methods available and unavailable for use in this study. Cut-off values used as the threshold for predicting sepsis or poor patient outcome in this study. BPM – beats per minute, GCS – Glasgow Coma Scale, HR – Heart Rate, RR – Respiratory Rate, WCC – White Cell Count.

For peer review only

For peer review only - http://bmjopen.bmj.com/site/about/guidelines.xhtml

Supplementary Table 3:

Outcome Measure	*Test*	*PPV (%)*	*NPV (%)*	*Sensitivity (%)*	*Specificity (%)*
Escalated care					
	SIRS	55.6 (39.3 to 71.8)	80.8 (72.0 to 89.5)	57.1 (40.7 to 73.5)	79.7 (70.9 to 88.6)
	NICE High-Risk	40.4 (27.0 to 53.7)	77.4 (67.0 to 87.8)	60.0 (43.8 to 76.2)	60.8 (50.0 to 71.5)
	Nice Moderate- and High-Risk	31.0 (21.9 to 40.1)	71.4 (47.8 to 95.1)	88.6 (78.0 to 99.1)	12.7 (5.3 to 20.0)
	qSOFA	35.0 (24.5 to 45.5)	79.4 (65.8 to 93.0)	80.0 (66.7 to 93.3)	34.2 (23.7 to 44.6)
	NEWS >4	53.3 (35.5 to 71.2)	77.4 (68.4 to 86.3)	45.7 (29.2 to 62.2)	82.3 (73.9 to 90.7)
30-Day Mortality					
	SIRS	19.4 (6.5 to 32.4)	92.3 (86.4 to 98.2)	53.8 (26.7 to 80.9)	71.3 (62.5 to 80.1)
	NICE High-Risk	13.5 (4.2 to 22.7)	90.3 (83.0 to 97.7)	53.8 (26.7 to 80.9)	55.4 (45.8 to 65.1)
	Nice Moderate- and High-Risk	13.0 (6.4 to 19.6)	100.0 (100.0 to 100.0)	100.0 (100.0 to 100.0)	13.9 (7.1 to 20.6)
	qSOFA	13.8 (6.2 to 21.3)	94.1 (86.2 to 100.0)	84.6 (65.0 to 100.0)	31.7 (22.6 to 40.8)
	NEWS >4	20.0 (5.7 to 34.3)	91.7 (85.8 to 97.6)	46.2 (19.1 to 73.3)	76.2 (67.9 to 84.5)
30-Day Admission					
	SIRS	88.9 (78.6 to 99.2)	32.1 (21.7 to 42.4)	37.6 (27.3 to 47.9)	86.2 (73.7 to 98.8)
	NICE High-Risk	80.8 (70.1 to 91.5)	30.6 (19.2 to 42.1)	49.4 (38.8 to 60.0)	65.5 (48.2 to 82.8)
	Nice Moderate- and High-Risk	75.0 (66.5 to 83.5)	28.6 (4.9 to 52.2)	88.2 (81.4 to 95.1)	13.8 (1.2 to 26.3)
	qSOFA	77.5 (68.3 to 86.7)	32.4 (16.6 to 48.1)	72.9 (63.5 to 82.4)	37.9 (20.3 to 55.6)
	NEWS >4	93.3 (84.4 to 100.0)	32.1 (22.2 to 42.1)	32.9 (22.9 to 42.9)	93.1 (83.9 to 100.0)
30-Day Composite					
	SIRS	88.9 (78.6 to 99.2)	30.8 (20.5 to 41.0)	37.2 (27.0 to 47.4)	85.7 (72.8 to 98.7)
	NICE High-Risk	80.8 (70.1 to 91.5)	29.0 (17.7 to 40.3)	48.8 (38.3 to 59.4)	64.3 (46.5 to 82.0)
	Nice Moderate- and High-Risk	76.0 (67.6 to 84.4)	28.6 (4.9 to 52.2)	88.4 (81.6 to 95.1)	14.3 (1.3 to 27.2)
	qSOFA	77.5 (68.3 to 86.7)	29.4 (14.1 to 44.7)	72.1 (62.6 to 81.6)	35.7 (18.0 to 53.5)
	NEWS >4	93.3 (84.4 to 100.0)	31.0 (21.1 to 40.8)	32.6 (22.7 to 42.5)	92.9 (83.3 to 100.0)

Legend:

Analysis of Scoring Systems vs outcome measures in patients >85 years old with suspected infection (n=114). PPV = Positive predictive value, NPV = negative predictive value. PPV, NPV, Sensitivity, and Specificity show percentage and 95% confidence intervals.

For peer review only - http://bmjopen.bmj.com/site/about/guidelines.xhtml

Supplementary Figure 1. ROC Curves showing the discriminating ability of the different sepsis scoring systems for escalated care for infection

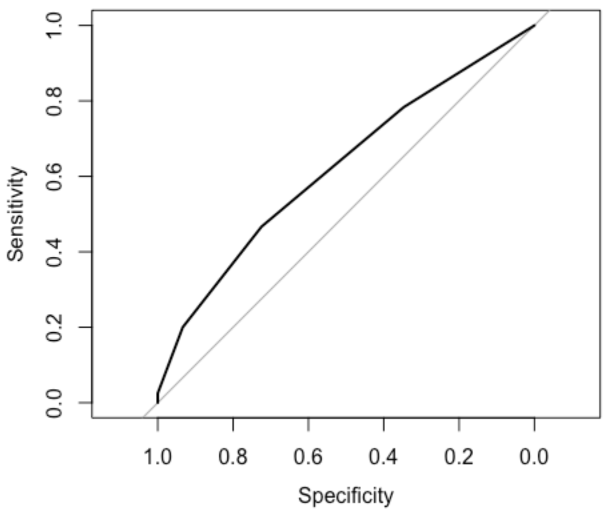

1a SIRS Area Under Curve (AUC) = 0.623 (95% CI: 0.5607-0.6843)

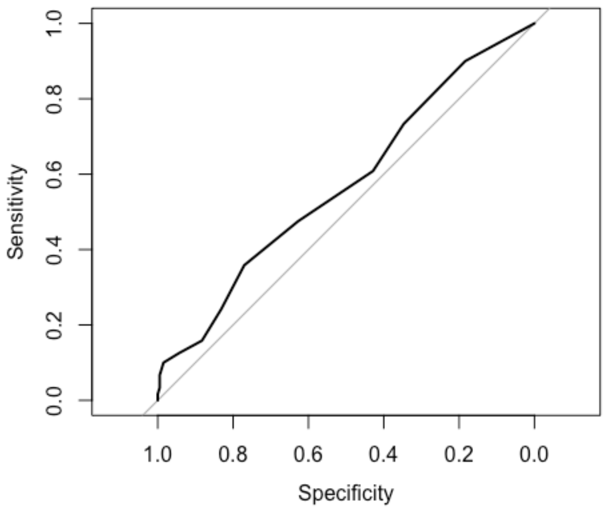

1b NEWS AUC = 0.573 (95% CI: 0.5079-0.6375)

For peer review only

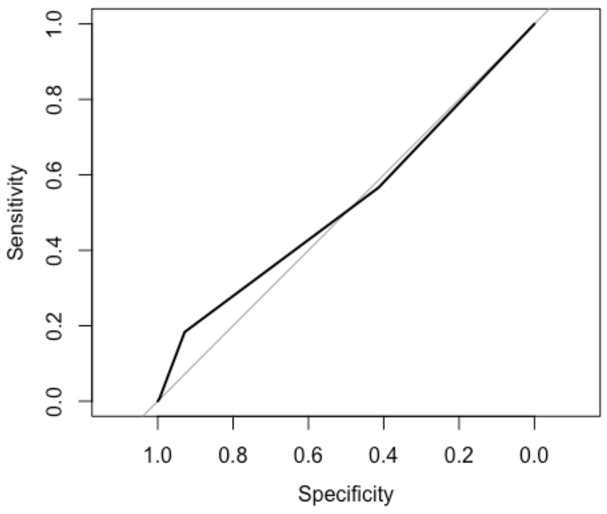

1c qSOFA AUC = 0.523 (95% CI: 0.4612-0.5855)

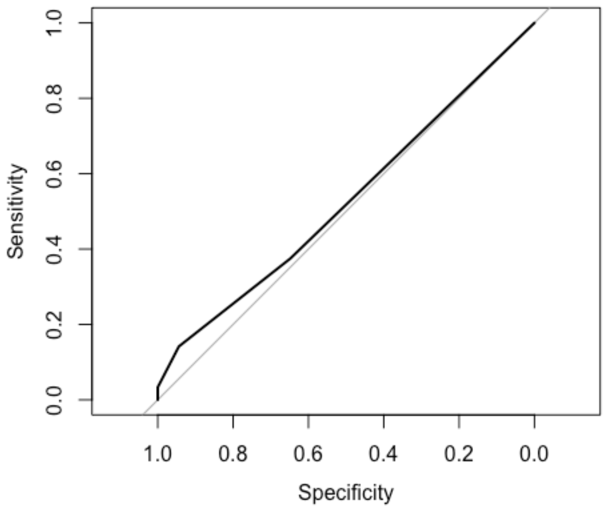

1d NICE High Risk AUC = 0.527 (95% CI: 0.4696-0.584)

For peer review only - http://bmjopen.bmj.com/site/about/guidelines.xhtml

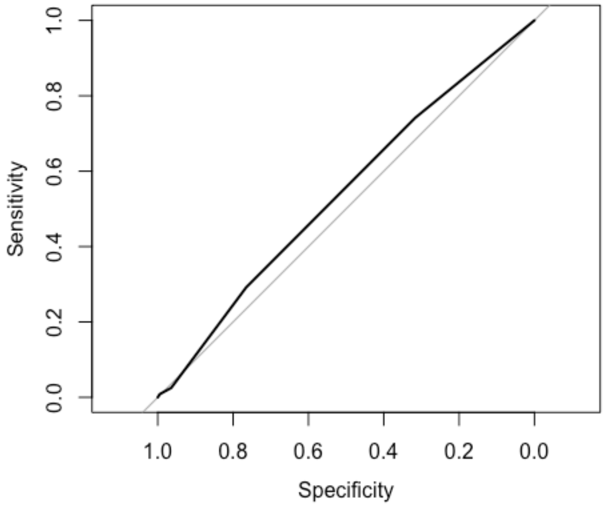

1e NICE Moderate and High Risk AUC = 0.540 (95% CI: 0.4784-0.6006)

For peer review only - http://bmjopen.bmj.com/site/about/guidelines.xhtml

STROBE Statement—Checklist of items that should be included in reports of ***cohort studies***

	Item No	Recommendation
**Title and abstract**	1	(*a*) Indicate the study's design with a commonly used term in the title or the abstract<br>P1
		(*b*) Provide in the abstract an informative and balanced summary of what was done and what was found<br>P2
**Introduction**		
Background/rationale	2	Explain the scientific background and rationale for the investigation being reported<br>P4
Objectives	3	State specific objectives, including any prespecified hypotheses<br>P5
**Methods**		
Study design	4	Present key elements of study design early in the paper<br>P5
Setting	5	Describe the setting, locations, and relevant dates, including periods of recruitment, exposure, follow-up, and data collection<br>P6
Participants	6	(*a*) Give the eligibility criteria, and the sources and methods of selection of participants. Describe methods of follow-up<br>P6
		(*b*) For matched studies, give matching criteria and number of exposed and unexposed<br>N/A
Variables	7	Clearly define all outcomes, exposures, predictors, potential confounders, and effect modifiers. Give diagnostic criteria, if applicable<br>P6
Data sources/ measurement	8*	For each variable of interest, give sources of data and details of methods of assessment (measurement). Describe comparability of assessment methods if there is more than one group<br>P6
Bias	9	Describe any efforts to address potential sources of bias<br>P5
Study size	10	Explain how the study size was arrived at<br>P5
Quantitative variables	11	Explain how quantitative variables were handled in the analyses. If applicable, describe which groupings were chosen and why<br>P6
Statistical methods	12	(*a*) Describe all statistical methods, including those used to control for confounding<br>P7
		(*b*) Describe any methods used to examine subgroups and interactions<br>P7 and supplementary material
		(*c*) Explain how missing data were addressed<br>P7
		(*d*) If applicable, explain how loss to follow-up was addressed<br>N/A
		(*e*) Describe any sensitivity analyses<br>P7 and supplementary material

For peer review only - http://bmjopen.bmj.com/site/about/guidelines.xhtml

For peer review only

**Results**		
Participants	13*	(a) Report numbers of individuals at each stage of study—eg numbers potentially eligible, examined for eligibility, confirmed eligible, included in the study, completing follow-up, and analysed<br>P7 and figure 1
		(b) Give reasons for non-participation at each stage<br>P7 and figure 1
		(c) Consider use of a flow diagram<br>Figure 1
Descriptive data	14*	(a) Give characteristics of study participants (eg demographic, clinical, social) and information on exposures and potential confounders<br>P7 and Table 1
		(b) Indicate number of participants with missing data for each variable of interest<br>Table 1
		(c) Summarise follow-up time (eg, average and total amount)<br>Table 1
Outcome data	15*	Report numbers of outcome events or summary measures over time<br>Table 2 reports patients where we had follow up to 30 days (set follow up time for the evaluation)
Main results	16	(*a*) Give unadjusted estimates and, if applicable, confounder-adjusted estimates and their precision (eg, 95% confidence interval). Make clear which confounders were adjusted for and why they were included<br>P7, P8 Table 2, Table 3, supplementary material
		(*b*) Report category boundaries when continuous variables were categorized<br>N/A
		(*c*) If relevant, consider translating estimates of relative risk into absolute risk for a meaningful time period<br>N/A
Other analyses	17	Report other analyses done—eg analyses of subgroups and interactions, and sensitivity analyses<br>P7, P8, Supplementary material
**Discussion**		
Key results	18	Summarise key results with reference to study objectives<br>P8
Limitations	19	Discuss limitations of the study, taking into account sources of potential bias or imprecision. Discuss both direction and magnitude of any potential bias<br>P9
Interpretation	20	Give a cautious overall interpretation of results considering objectives, limitations, multiplicity of analyses, results from similar studies, and other relevant evidence<br>P9
Generalisability	21	Discuss the generalisability (external validity) of the study results<br>P 10
**Other information**		
Funding	22	Give the source of funding and the role of the funders for the present study and, if applicable, for the original study on which the present article is based<br>P11

For peer review only

*Give information separately for exposed and unexposed groups.

Note: An Explanation and Elaboration article discusses each checklist item and gives methodological background and published examples of transparent reporting. The STROBE checklist is best used in conjunction with this article (freely available on the Web sites of PLoS Medicine at http://www.plosmedicine.org/, Annals of Internal Medicine at http://www.annals.org/, and Epidemiology at http://www.epidem.com/). Information on the STROBE Initiative is available at http://www.strobe-statement.org.

For peer review only

For peer review only - http://bmjopen.bmj.com/site/about/guidelines.xhtml
